# A refactored biosynthetic pathway for the production of glycosylated microbial sunscreens†

**DOI:** 10.1039/d4cb00128a

**Published:** 2024-08-20

**Authors:** Sıla Arsın, Maija Pollari, Endrews Delbaje, Jouni Jokela, Matti Wahlsten, Perttu Permi, David Fewer

**Affiliations:** a Department of Microbiology, Faculty of Agriculture and Forestry, University of Helsinki 00014 Helsinki Finland david.fewer@helsinki.fi; b Faculdade de Ciências Farmacêuticas de Ribeirão Preto, Universidade de São Paulo Ribeirão Preto Brazil; c Department of Agricultural Sciences, Faculty of Agriculture and Forestry, University of Helsinki 00014 Helsinki Finland; d Department of Chemistry, University of Jyväskylä 40014 Jyväskylä Finland; e Department of Biological and Environmental Science, Nanoscience Center, University of Jyväskylä 40014 Jyväskylä Finland

## Abstract

Mycosporine-like amino acids (MAAs) are a family of water-soluble and colorless secondary metabolites, with high extinction coefficients, that function as microbial sunscreens. MAAs share a cyclohexinimine chromophore that is diversified through amino acid substitutions and attachment of sugar moieties. The genetic and enzymatic bases for the chemical diversity of MAAs remain largely unexplored. Here we report a series of structurally distinct MAAs and evidence for an unusual branched biosynthetic pathway from a cyanobacterium isolated from lake sediment. We used a combination of high-resolution liquid chromatography-mass spectrometry (HR-LCMS) analysis and nuclear magnetic resonance (NMR) spectroscopy to identify diglycosylated-palythine-Ser (C_22_H_36_N_2_O_15_) as the dominant chemical variant in a series of MAAs from *Nostoc* sp. UHCC 0302 that contained either Ser or Thr. We obtained a complete 9.9 Mb genome sequence to gain insights into the genetic basis for the biosynthesis of these structurally distinct MAAs. We identified MAA biosynthetic genes encoded at two locations on the circular chromosome. Surprisingly, direct pathway cloning and heterologous expression of the complete *mysABCJ*_*1*_*D*_*1*_*G*_*1*_*H* biosynthetic gene cluster in *Escherichia coli* (*E. coli*) led to the production of 450 Da monoglycosylated-palythine-Thr (C_18_H_30_N_2_O_11_). We reconstructed combinations of the two distant biosynthetic gene clusters in refactored synthetic pathways and expressed them in the heterologous host. These results demonstrated that the MysD_1_ and MysD_2_ enzymes displayed a preference for Thr and Ser, respectively. Furthermore, one of the four glycosyltransferases identified, MysG_1_, was active in *E. coli* and catalysed the attachment of a hexose moiety to the palythine-Thr intermediate. Together these results provide the first insights into the enzymatic basis for glycosylation of MAAs and demonstrates how paralogous copies of the MysD enzymes allow the simultaneous biosynthesis of specific chemical variants to increase the structural variation in this family of microbial sunscreens.

## Abstract 2

MAA biosynthetic enzymes encoded in two distant sections of the genome work together to form two diglycosylated MAA structural variants.

## Graphical abstract

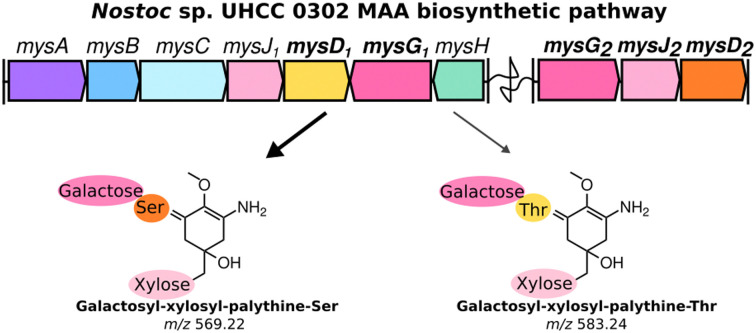

## Introduction

Photoautotrophic organisms, such as cyanobacteria, have developed mechanisms to counteract the damaging effects of UV radiation by producing protective pigments and secondary metabolites.^1^ Among these secondary metabolites, mycosporine-like amino acids (MAAs) serve as efficient sunscreens, with high molar extinction coefficients of up to 50 000 M cm^−1^, effectively absorbing UVA/UVB radiation.^2^ MAAs can dissipate 98% of the absorbed radiation as heat without generating radical oxygen species and some MAA variants were even shown to possess potent radical scavenging properties.^3–6^ Given their non-toxic nature, efficient UV absorption capabilities, antioxidant potency and potential other anti-inflammatory and anti-aging activities MAAs have garnered significant attention in the scientific community over the past decade, showcasing their potential applications in the cosmetic and pharmaceutical industries.^7–11^

MAAs are small, colorless, water-soluble compounds composed of either a cyclohexenone or cyclohexenimine chromophore, with amino acid substituents attached to the first carbon (C1) or third carbon (C3).^1,12^ The structural diversity of MAAs can be further expanded through modifications in the amino acid residues and glycosylation.^13–15^ Shinorine (absorption maximum (*λ*_max_) = 333 nm, 333 Da) stands out as one of the most prevalent MAA variants in cyanobacteria, and its biosynthetic pathway involving the *mysABCD/E* gene cluster was the first to be elucidated.^16–18^ Cyanobacterial MAA biosynthesis seems to be able to either involve both the shikimate and pentose phosphate pathways or to be strictly dependent on one pathway to supply the precursor sugar–phosphate.^19–22^ In the established biosynthetic pathway, MysA functions as 2-*epi*-5-*epi*-valiolone synthase (EEVS), which acts on sedoheptulose 7-phosphate from the pentose phosphate pathway forming 2-*epi*-5-*epi*-valiolone intermediate that gets methylated by a *O*-methyltransferase enzyme (MysB) to make the 4-deoxygadusol core.^16^ Following this step, MysC, an ATP-grasp ligase, catalyzes the addition of Gly onto the C1 position, resulting in the formation of mycosporine-glycine.^16,17^ The addition of Ser onto the C3 position is carried out either by MysE, a non-ribosomal peptide synthetase (NRPS), or by MysD, recently renamed as mycosporine-glycine amine-ligase (MGA ligase) as recently proposed.^16,23^ MysD was shown to exhibit a level of substrate promiscuity with higher affinities towards Ser or Thr.^24^ The phytanoyl-CoA dioxygenases, MysH, have also recently been identified as important in the biosynthesis of palythine (*λ*_max_ = 320 nm, 244 Da) variants.^24^ In our previous work we have also noted the presence of more complex MAA biosynthetic pathways and presented additional potential substrates for MysE enzymes as well as a role for methyltransferase enzymes (MysF) in aplysiapalythine-type MAA synthesis.^25^

Numerous glycosylated MAA structural variants have also been reported in cyanobacteria, particularly in the genus *Nostoc*. However, the glycosyltransferase enzymes responsible for the addition of sugar moieties remained unidentified.^13,14,25,26^ Here, we investigated the MAA biosynthetic pathway of the benthic *Nostoc* sp. UHCC 0302, maintained at the University of Helsinki Culture Collection (UHCC). *Nostoc* sp. UHCC 0302 constitutively produces 568 Da diglycosylated; hexosyl-pentosyl-palythine-Ser as the major structural variant. We provide a complete genome for *Nostoc* sp. UHCC 0302 that contains a type of discontiguous MAA biosynthetic gene cluster which includes a pair of two distinct glycosyltransferase enzymes as well as a pair of MysD homologues. Through heterologous expression experiments involving reconstructed MAA biosynthetic gene clusters in *E. coli*, we have demonstrated potential involvement of two different glycosyltransferase enzymes in MAA glycosylation. Additionally, our results provide further insights into the distribution of various MysD homologs involved in MAA synthesis, each exhibiting distinct substrate specificities that contribute to the structural diversity of MAAs.

## Results & discussion

### Structural MAA variants of *Nostoc* sp. UHCC 0302

**Fig. 1:**
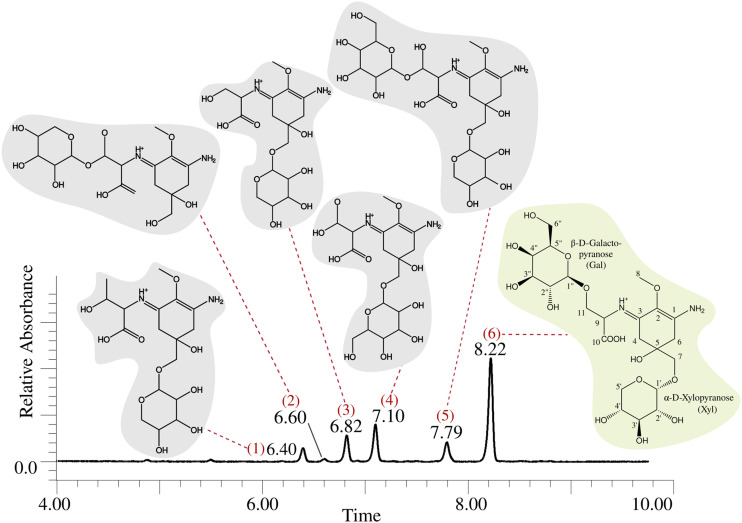
MAA chemical variants of *Nostoc* sp. UHCC 0302 detected by HR-LCMS. The predicted chemical structures of the MAA variants are shown corresponding to their peak of absorbance at 320 nm. The dominant, 568 Da, 11-(β-d-galactopyranosyl)-7-(α-d-xylopyranosyl)-palythine-Ser chemical structure is highlighted in light green background and its chemical structure is based on the NMR analysis (Tables S2, S3 and Fig. S2–S19, ESI†).

We discovered the MAA production in *Nostoc* sp. UHCC 0302 by HR-LCMS-UV analysis of the dried biomass extracts according to the absorbance peaks at 320 nm (Fig. 1 and Fig. S1, ESI†). Based on these results, *Nostoc* sp. UHCC 0302 constitutively produces two diglycosylated variants and four monoglycosylated MAA structural intermediates (Fig. 1 and Table S1, ESI†). The main MAA variant made up 52.1% of the total area of absorbance with *m*/*z* of 569.22 and retention time of 8.22 minutes, corresponding to a palythine-Ser with hexose and pentose moieties (Table S1 and Fig. S1, ESI†). Product ions of protonated 568 Da main MAA MS^E^ spectrum fitted perfectly to this structure (Fig. S2 and Table S2, ESI†). The second diglycosylated MAA structural variant (no. 5) made up only 10.4% of the total absorbance and had a *m*/*z* of 583.24 at 7.79 minutes, corresponding to a palythine-Thr with a hexose and a pentose moiety (Table S1 and Fig. S1, ESI†). The peaks 1–4 are the monoglycosylated structural intermediates for the two main MAA variants No 5. and 6. (Table S1 and Fig. S1, ESI†).

We purified the major MAA chemical variant (no. 6) with absorbtion maximum at 323 nm, C_22_H_36_N_2_O_15_ (*m*/*z* 569.21945 for [M + H]^+^, Δ 1.1 ppm) for further characterization with NMR analysis (Fig. 1 and Fig. S11, Table S1, ESI†). The sample was dissolved to D_2_O and ^1^H, ^1^H–^1^H DQF–COSY, ^1^H–^1^H TOCSY, ^1^H–^13^C HSQC–TOCSY, CH_*n*_ multiplicity edited ^1^H–^13^C HSQC and ^1^H–^13^C HMBC spectra were measured. Numerical data is presented in Table S3 (ESI†) and spectra in Fig. S3–S8 (ESI†). All δ_H_ and δ_C_ signals and COSY/HMBC correlations for atom positions 1–11 were typical for the palythine-Ser (PS) structure (Table S4 and Fig. S11, ESI†). Also, resonances from one pentose (Pent) and one hexose (Hex) were recognized. HMBC correlation between Hex anomeric H-1 and PS C-11 was present which means that Hex is connected to the PS unit 11-OH (Fig. 1 and Fig. S9, ESI†). Hex had coupling constant ^3^*J*_H1,H2_ of 8.0 Hz meaning that both H1 and H2 are axial which is the situation in β-d-glucose, -galactose, -gulose and -allose. A correlation from Pent anomeric H-1 and PS C-7 was present which means that Pent is connected to the PS unit 7-OH (Fig. 1 and Fig. S9, ESI†). All δ_H_ and δ_C_ signals and vicinal coupling constant ^3^*J*_H1,H2_ of 3.7 Hz match best to d-pentoses with α-d-xylopyranose structure. Data from proton spectrum of hydrolyzed 568 Da MAA confirmed that subunit monosaccharides were galactose (Gal) and xylose (Xyl) (Fig. S10, ESI†). Both Gal and Xyl C-1 signals appear at region typical for O-linked glycosides (Agrawal Phytochem 1992). The PS unit C-7 δ_C_ signal had shifted 5 ppm downfield compared to average data from –(C-7) H_2_OH from La Barre and colleagues which also shows that C-7 OH is not free but bonded.^27^

### Refactored expression of the main MAA biosynthetic gene cluster of *Nostoc* sp. UHCC 0302 in *Escherichia coli* BL21 (DE3)

**Fig. 2:**
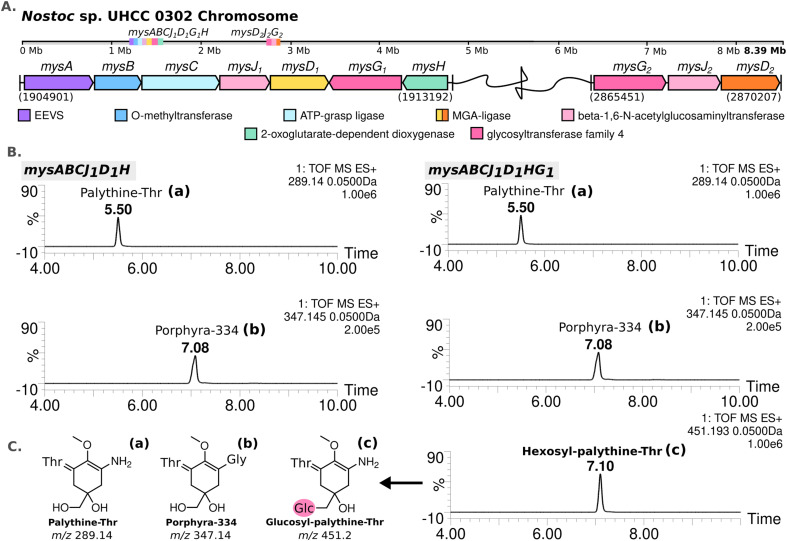
MAA biosynthetic gene clusters detected in the complete genome of *Nostoc* sp. UHCC 0302 (A). MS-ES+ chromatograms of the detected MAA variants from *E. coli* BL21 (DE3) transformants expressing the main MAA biosynthetic gene cluster with and without *mysG* in pET28a+ system (B) with the simplified structures of the variants detected, labelled as palythine-Thr (a), porphyra-334 (b) and hexosyl-palythine-Thr (7-(α-d-glucopyranosyl)-palythine-Thr) based on NMR data (Tables S5, S6 and Fig. S11–S17, ESI†) (C).

The complete genome assembled for *Nostoc* sp. UHCC 0302 (CP151099) was 9 859 258 bp and comprised of an 8 388 664 bp circular chromosome and 8 plasmids. Based on the known MAA biosynthetic enzyme sequences in the literature, we screened the genome of *Nostoc* sp. UHCC 0302 and identified the potential MAA biosynthetic gene clusters. As anticipated, we found an 8.3 kb gene cluster which included the well-established four MAA biosynthetic genes (*mysABCD)* and an additional three, designated as *mysABCJ*_*1*_*D*_*1*_*G*_*1*_*H* (Fig. 2(A)). MysH is homologous to 2-oxoglutarate-dependend dioxygenase, recently shown to be involved in palythine synthesis.^24^ MysJ_1_ and MysG_1_ are glycosyltransferase enzymes homologous to beta-1-1,6-*N*-acetylglucosaminyltransferase and glycosyltransferase family 4 proteins respectively (Fig. 2(A)). Additionally, we identified and annotated a distant and incomplete, *mysD*_*2*_*J*_*2*_*G*_*2*_ enzyme cluster for their potential involvement in MAA biosynthesis in *Nostoc* sp. UHCC 0302 (Fig. 2(A)).

We conducted heterologous expression experiments to elucidate the roles of enzymes involved in the biosynthesis of MAA structural variants detected in *Nostoc* sp. UHCC 0302. Initially, we designed a construct containing only the main (complete) MAA biosynthetic gene cluster *mysABCJ*_*1*_*D*_*1*_*G*_*1*_*H* in the pET28a+ vector for heterologous expression in *E. coli*. MAA structural variants produced by these clones were analysed by HR-LCMS screening of their methanol extracts (Fig. S18, ESI†). Here, we detected *m*/*z* 451 [M + H]^+^ hexosyl-palythine-Thr as the main MAA product which is direct match to the intermediate no 4 detected in *Nostoc* sp. UHCC 0302 (Fig. 1 and 2(B), (C)). Following this, we confirmed the structure of the purified hexosyl-palythine-Thr as 7-(α-d-glucopyranosyl)-palythine-Thr using NMR (Fig. S11–S17, ESI†). The intermediates of porphyra-334 and palythine–threonine were still detected in comparable amounts alongside the main hexose bound variant (Fig. 1 and 2(B)). This could be due to various reasons such as suboptimal protein expression and folding, differing substrate availabilities, pathway flux and/or potential metabolic interferences in *E. coli.*^28^

Next, we wanted to determine which glycosyltransferase is responsible for the hexose addition, for this we omitted the *mysG*_*1*_ gene and tested the expression of construct *mysABCJ*_*1*_*D*_*1*_::pET28a+ (Fig. 2(B) and Fig. S18, ESI†). The heterologous expression of the constructs including only MysJ_1_, resulted in agluconic structural variants of palythine-Thr and porphyra-334 (Fig. 2(B) and Fig. S18, ESI†). This revealed that, *mysG*_*1*_ annotated as glycosyltransferase family 4, is responsible for the attachment of a hexose onto the MAA variants (Fig. 2(B)). BlastP screening of both MysG_1/2_ amino acid sequences also show similarity with WcaC-type enzymes which are involved in addition of sugar units in colanic acid biosynthesis.^29^ Colanic acid is composed of repeating hexasaccharide chains as part of the protective exopolysaccharide (EPS) layer.^30^ We think MysG_1/2_ may also function in a similar way in EPS synthesis and also MAA glycosylation. Presence of MAAs in analysed EPS extracts and synergistical upregulation of both EPS and MAAs pathways in response to UV and desiccation stress could also be further evidence for localisation of glycosylated MAAs in EPS layers.^13,14,31,32^ This incorporation may help these organisms develop an effective outer shield against UV exposure and dessication.

Conversely, we observed no activity for MysJ_1_ (a beta-1,6-*N*-acetylglucosaminyltransferase enzyme) or for the other glycosyltransferases encoded in the distant *mysD*_*2*_*J*_*2*_*G*_*2*_ cluster in our expression systems (Fig. 2(B) and Fig. S18, ESI†). In *Nostoc* sp. UHCC 0302, MysJ_1_ is likely responsible for adding a pentose sugar, resulting in a variant corresponding to the 568 Da diglycosylated palythine Ser (no. 6) (Fig. 1 and 2). It is probable that the glycosylation of MAAs for potential incorporation into EPS layer involves a complex series of reactions by several glycosyltransferase enzymes, an activity likely also not supported in our *E. coli* system.^33^ Our additional homology-based protein model and binding prediction revealed that both MysJ_1/2_ and MysG_1/2_ type enzymes are not well characterized as the best hits with highest sequence identity provided minimum to no information (Table S11, ESI†). Interestingly for MysJ_1/2_ enzymes, the second best hit with around 30% sequence identity is a xylosyltransferase 1 enzyme found in human genomes with established activity in catalysing xylose addition onto Ser and Thr residues (Table S11, ESI†).^34^ Similarly the second best hit for MysG_1/2_ were of low sequence identity (∼21.5%), but a well characterized sucrose–phosphate enzyme from *Thermosynechococcus elongatus* with suggested glucose transfer activity onto a fructose-6-phospahate (Table S11, ESI†).^35^

We then investigated the distribution of these glycosyltransferase enzymes identified in *Nostoc* sp. UHCC 0302, in 336 complete publicly available cyanobacterial genomes. Among these genomes which included a type of an MAA biosynthetic gene cluster, 52 strains contained glycosyltransferase enzymes homologous to MysG_1/2_ and MysJ_1/2_ based on 40% minimum alignment identity and coverage. While the majority (87%) of these genomes encoded glycosyltransferases homologous to MysG, only 7 (13%) genomes encoded enzymes with homology to MysJ, forming a single cluster (Fig. S21, ESI†). These were *Nostoc* sp. ATCC 53789, *Nostoc* sp. *Lobaria pulmonaria* 5183 cyanobiont, *Nostoc* sp. NIES-4103, *Nostoc linckia* NIES-25, *Nostoc* sp. C052 and our *Nostoc* sp. UHCC 0302 (Fig. S21, ESI†). It is noteworthy that all MysJ encoding genomes were exclusively found within the *Nostoc* genus and co-encoded a MysG enzyme (Fig. S21, ESI†). This observation suggests a potential interdependence between MysJ and MysG, with MysJ involvement being rarer and specific to *Nostoc* species (Fig. S21, ESI†). In addition, *Nostoc* sp. UHCC 0302 seems the be unique among the studied strains as it encodes as MysJ_1/2_ and MysG_1/2_ (Fig. S21, ESI†). Cyanobacterial genomes encode multiple glycosyltransferases which are generally thought to be mainly involved in the synthesis of complex EPS layers, yet the activities of these enzymes are scarcely understood.^36,37^ To confirm the exact activities of MysJ_1/2_ and MysG_1/2_ further work involving structural characterisation and activity assays are necessary. Our analysis thus far can only predict that both sets of enzymes might have a role in MAA glycosylation based on the identified MAA variants in *Nostoc* sp. UHCC 0302, experiments and bioinformatic analysis.

### MysD1 and MysD2 have different substrate preferences

**Fig. 3:**
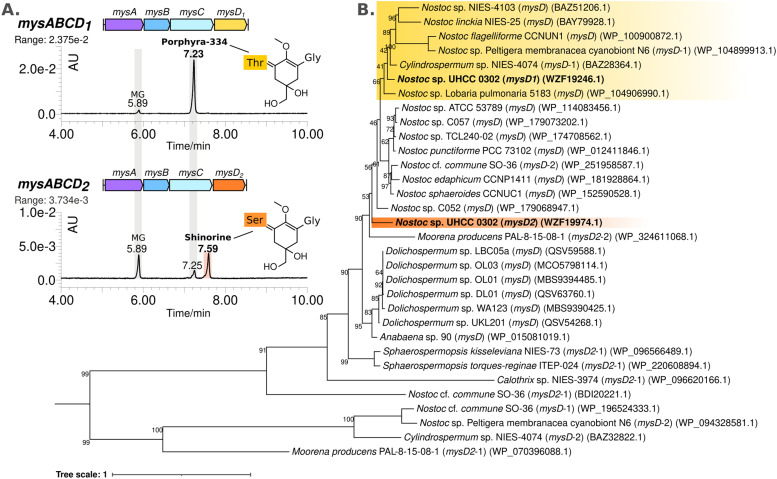
Comparing MAA intermediates and chemical variants detected by HR-LCMS-UV absorbance peak at 320 nm, extracted from *E. coli* BL21 (DE3) colonies carrying the synthetic MAA biosynthetic gene cluster constructs *mysABCD*_*1*_, and *mysABCD*_*2*_ in pBAD/HISB expression system (A). Phylogenetic distribution of the MysD enzymes selected from complete cyanobacterial genomes encoding for multiple MysD homologs in and near MAA biosynthetic gene clusters constructed using with IQ-TREE v1.7 default parameters and substitution models JTTDCMut + G4 (B).

**Fig. 4:**
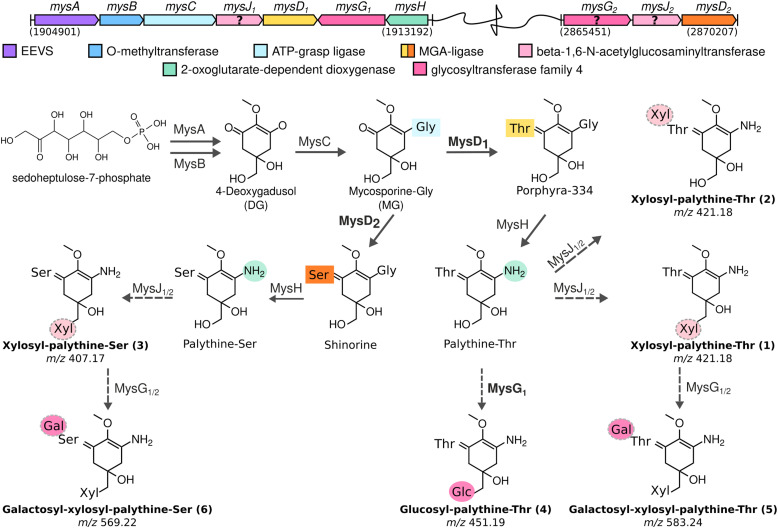
MAA biosynthetic gene clusters identified in the chromosome of *Nostoc* sp. UHCC 0302 and the proposed biosynthetic scheme based on heterologous expression results for MAA chemical variants and intermediates detected by HR-LCMS. Detected MAA chemical structures are numbered to match the HR-LCMS structures on Table S1 (ESI†). (Galactose: Gal, glucose: Glc, xylose: Xyl).

The MAA structural variants produced by the recombinant *E. coli* BL21 (DE3) are almost exclusively the Thr bound, instead of Ser, which is the dominant structural variant we detect from *Nostoc* sp. UHCC 0302 samples (Fig. 2 and Table S1, ESI†). To test whether the additional set of enzymes encoded in the distant 4.8 kb cluster of *mysD*_*2*_*J*_*2*_*G*_*2*_, are also involved in MAA synthesis, the *mysABCD*_*1*_*D*_*2*_*H* gene cluster was expressed in *E. coli*, using the araBAD system which allows for tight regulation of expression. We modified this initial construct with restriction enzymes to also obtain the constructs *mysABCD*_*1*_ and *mysABCD*_*2*_ to compare the different MAA structural variants produced by each individual MysD homolog. Recombinant *E. coli* with the gene cluster including MysD_1_ produced almost exclusively Thr-bound variants while the version with MysD_2_ dominantly produced Ser-bound variants (Fig. 3(A)). In addition, small amounts of Ala- and Thr- bound variants were detected (Fig. 4 and Fig. S20, ESI†). Previous literature proposes that all MGA-ligases are generally promiscuous,^23,24^ and while MysD_1_ and MysD_2_ homologues share high sequence identity (84.35%), their substrate preferences and levels of promiscuity clearly vary. Recently, Kim and colleagues demonstrated that 43 amino acids forming the omega loop structure in MysD enzymes determine the substrate specificity, meaning there are different MysD derivatives.^38^ In agreement to this, in our work we can also see that MysD_1_ is highly specific for Thr and MysD_2_ is more flexible, catalyzing additions of Ala and Thr in small amounts while having a stronger preference for Ser (Fig. S20, ESI†).

We observed a scattered phylogenetic distribution amongst MysD enzymes identified from 27 cyanobacterial genomes encoding multiple copies of MysD enzymes near MAA biosynthetic gene clusters (Fig. 3(B)). Notably, in the case of *Nostoc* sp. UHCC 0302, MysD_2_ appears to represent an earlier evolutionary iteration compared to MysD_1_, grouping within a clade predominantly consisting of *Nostoc* sp., some of which are known to produce tricore MAAs (Fig. 3(B)).^25^ Promiscuity is regarded as part of enzymatic evolution and such substrate flexibility in secondary metabolite synthesis tends to be favoured as it enhances structural diversity of the metabolites contributing to fitness of the organisms in a changing environment.^39–41^ The phenomenon is widespread in nature and these shifts between promiscuity and specificity can occur by mere point mutation, leading to cases such as this one.^40,42^ In *Nostoc* sp. UHCC 0302 the presence of different two distinct and active MysD homologs with varying substrate specificities is likely due to series of horizontal gene transfer events. Interestingly, the distant MysD_2_ is the dominant active enzyme in *Nostoc* sp. UHCC 0302 as the major MAA variant is the Ser bound, galactosyl-xylosyl-palythine-Ser (no. 6) (Fig. 1). The cooperation of enzymes from discontiguous biosynthetic gene clusters is reported amongst MAA biosynthetic pathways^25^ and such cooperation might be one of the key mechanisms resulting in structural MAA variants with a seemingly simple and conserved MAA biosynthetic gene cluster.

### Proposed biosynthetic pathway for the MAA structural variants of *Nostoc* sp. UHCC 0302

Based on the results of our heterologous expression of the refactored MAA biosynthetic gene clusters of *Nostoc* sp. UHCC 0302 in *E. coli* BL21 (DE3) we propose a biosynthetic scheme for the different MAA structural variants detected as presented on Fig. 1. MysA, MysB, MysC and MysH enzymes work as previously established in *E. coli* as well, as upon their inclusion in our constructs, we consistently detected mycosporine-glycine and palythine variants in our heterologous expression experiments (Fig. S18 and S20, ESI†).^16,17,24^ In addition, we also show the involvement of the distant and partial MAA biosynthetic gene cluster *mysD*_*2*_*F*_*2*_*G*_*2*_, in the biosynthesis of at least the dominant 568 Da galactosyl-xylosyl-palythine-Ser (no. 6) variant (Fig. 4). While the MysD_1_ enzyme of the main MAA biosynthetic gene cluster mainly catalyses the addition of the Thr residue onto the C3 of MAA chromophore, the distantly encoded MysD_2_ is responsible for catalysing the addition of Ser residue, forming the palythine-Ser core of the major MAA variant (Fig. 4 and Table S1, ESI†). This finding mirrors the discontinuous MAA biosynthetic pathway previously hypothesized for *Nostoc* sp. UHCC 0926, suggesting potential collaboration between distant MAA biosynthetic gene clusters.^25^ Furthermore, our experiments implicate MysG_1_ in MAA biosynthesis, demonstrating its involvement in the addition of a hexose sugar, specifically glucopyranose, onto C7 of palythine-Thr, to generate glucosyl-palythine-Thr (no. 4) (Fig. 2 and 4). However, further investigation is warranted to fully elucidate rest of the glycosylation reactions shown in this scheme. To emphasize the gap in our understanding regarding these reactions involving MysJ_1_, MysJ_2_, MysG_2_, respective encoding genes are marked with a question mark and their suggested activities are depicted with dashed arrow lines in the biosynthetic scheme (Fig. 4). Based on our experimental and bioinformatic data, our hypothetical scheme assumes the addition of all other hexose–sugars are catalysed by MysG_1_ or MysG_2_, and xylose moieties by MysJ_1/2_ to generate the MAA structural variants we have detected (Fig. 1 and 4).

## Conclusions

The MAA biosynthetic enzymes found encoded in two distant locations on the genome of *Nostoc* sp. UHCC 0302 seem to work together to synthesize the novel diglycosylated, 11-(β-d-galactopyranosyl)-7-(α-d-xylopyranosyl)-palythine-Ser alongside the minor hexosyl-pentosyl-palythine-Thr variant. In this biosynthetic gene cluster, we identified two distinct pairs of glycosyltransferase enzymes, MysJ_1/2_ and MysG_1/2_ and demonstrated a potential activity for MysG_1_, in catalysing the addition of a glucose moiety to synthesise a glucosyl-palythine-Thr in *E. coli*. To our knowledge this is the first report linking a glycosyltransferase enzyme to MAA glycosylation. In addition, the study of active MysD homologs provided further insights into MGA ligase substrate specificities and diversity in cyanobacterial MAA biosynthesis.

This study reveals novel insights for the genetic and molecular mechanisms underlying the structural diversity of MAAs in cyanobacteria. Despite the apparent simplicity of MAA biosynthetic gene clusters, our findings suggest these gene cluster organisations diversified by constant reconfiguration, leading to the observed structural variation. In essence, our work not only advances our understanding of the genetic and molecular mechanisms governing the biosynthesis of different MAA variants but also underscores the dynamic nature of these pathways.

## Materials and methods

### Isolation and cultivation of *Nostoc* sp. UHCC 0302

*Nostoc* sp. UHCC 0302 was isolated on 16.02.1999, from the sediment surface of lake Hiidenvesi in Nummelanselkä, Finland (60°20’38.1’’N 24°17’07.8’’E; 60.34400302025203, 24.28525053485131). The axenic culture is maintained in 40 ml modified Z8 media^43^ under the light intensity of 8.70 μmol m^−1^ s^−1^. Mass cultivation of *Nostoc* sp. UHCC 0302 was achieved by growing the cultures in 40 L batches for 4 weeks at a time. To purify the dominant MAA chemical variant, we obtained 10 g dried cell biomass by harvesting the cultures *via* centrifugation using the Sorvall Lynx 6000 (Thermo Scientific) at 9000 g for 8 minutes at 20 °C and lyophilisation with the Christ LCS Plus Beta 2–8 LCS Plus Freeze Dryer at 0.0650 mbar for 48 hours.

### Extraction and detection of MAAs by HR-LCMS-UV

Approximately 50–100 mg of dried cell biomass was collected in 2 ml plastic screw-cap Eppendorf tubes filled with 200 μl of 0.55 mm Glass Micro Beads (Scientific Industries) were added to a screw-top Eppendorf tube. Cells were then disrupted in 1 ml of methanol at 6.5 m s^−1^ for 20 seconds using a Fast Prep-24 (MP Biomedicals™). Once lysed, the samples were centrifuged for 5 minutes at 10 000*g* using an Eppendorf centrifuge 5415D. 100 μl of the supernatant was taken up and filtered using Injekt-F 1 ml syringe (B-Braun) with 0.2 μm Fisherbrand PTFE syringe filter tip (Fisher Scientific) into a short thread sample vial (VWR) for HR-LCMS analysis.

An initial method of screening was performed using UPLC-QTOF (Acquity I-Class UPLC-SynaptG2-Si, Waters Corp., Milford, MA, USA) with the ACQUITY UPLC BEH Amide Column (2.1 mm × 100 mm, 1.7 μm, 130 Å, Waters Corp., Milford, MA) with solvent A: 0.2% ammonium formate and solvent B: acetonitrile with a flow rate of 0.300 ml min^-1^. The initial percentages of solvents were: 10% solvent A and 90% solvent B, which changed linearly to 40% solvent A and 60% solvent B by 9.00 minutes. The sample was injected 0.5 μl at a time. The target sample temperature was 5.0 °C and column temperature: 40.0 °C. Samples were run at ES^+^ polarity with the capillary voltage at 2.5 kV. The sampling cone was set to 20 V with a source temperature of 120 °C and desolvation temperature of 600 °C. The cone gas flow was set to 50 L h^−1^ and desolvation gas flow to 1000.0 L h^−1^ with a nebuliser gas flow of 6.0 bar. Photodiode array detector recorded between 210 to 800 nm.

### Purification of the 568 Da diglycosylated palythine-Ser from *Nostoc* sp. UHCC 0302

The most abundant diglycosylated MAA variant of *m*/*z* 569 detected from *Nostoc* sp. UHCC 0302 was purified for further characterization. For this, *Nostoc* sp. UHCC 0302 cells grown in liquid media were collected and freeze dried. Then, 1 gram of the dried sample material was dissolved in 30 ml of methanol and cells were broken down using Silent Crusher M (Heidolph) for 1 minute at 20 000*g* and centrifuged at 5000*g* for 10 minutes for three times. Supernatants were collected into a rotor evaporator flask and mixed with 10 ml of 100–200 mesh Chromatorex chromatography silica gel (Fuji-Davison Chemical). Methanol was evaporated from the sample using Vacuum Controller V-800 Rotavapor R-200 (Buchi) at 130 mbar at 30 °C. MAAs were then purified using a Strata Silica, Florisil, NH_2_, CN Normal Phase column (Strata) for polar retention mechanisms primed with 100% isopropanol and heptane. Dried cell material bound to silica gel was loaded as 5 ml at a time at the top of the column and eluted with 10 ml of 100% heptane, ethyl acetate, dichloromethane, acetone and methanol.

The methanol eluate was centrifuged and diluted in 100 × methanol to confirm the presence of mycosporines by measuring the samples in a UV-1800 Ordior spectrophotometer (Shimadzu) with the spectrum range of 190 to 400 nm. Methanol samples were dried using TurboVap LV Evaporator (Zymark) at 30 °C with pressure set to 0.4 bar, re-dissolved in 1 ml ultra-pure water, homogenized by vortexing for 10 seconds and sonicating with Sonorex Super 10P (Bandelin) for 10 seconds. Samples were then centrifuged at 10 000 g for 5 minutes and supernatants were collected to be fractioned using the high-performance liquid chromatography (HPLC) method. For each run, 50 μl samples were injected into the XSelect HSS T3 column (10 × 150 mm, 5 μm, Waters) in the HP Agilent 1100 series liquid chromatograph (Hewlett Packard) for reverse phase HPLC. The flow rate of 4.5 ml min^−1^ was set with 100% of 0.1% formic acid. Fractions were then detected using the UV-Vis diode array detector at the wavelength range of 200 to 330 nm.

### Structural characterisation of purified MAA variants

For the NMR spectroscopy based structural characterization, the samples of MAA chemical variants with MW of 568 and 450 Da, were dissolved in D_2_O followed by the collection of spectral data at 298 K. All NMR spectra were measured using a Bruker Avance III HD 800 MHz NMR spectrometer having a ^1^H, ^13^C, ^15^N triple resonance cryogenically cooled TCI probehead with a z-gradient coil.

In addition to one-dimensional ^1^H spectra, the structural characterization was accomplished using a panoply of two-dimensional (2D) homo- and heteronuclear NMR experiments. 2D ^1^H, ^1^H TOCSY (total correlation spectroscopy) experiment, using DIPSI-2 sequence with a mixing time (*t*_m_) of 90 ms was supplemented with 2D ^1^H,^1^H DQF–COSY (double quantum filtered correlation spectroscopy) for the spin-system identification. 2D ^1^H,^13^C HSQC and CH_*n*_-multiplicity edited ^1^H, ^13^C HSQC (heteronuclear single quantum coherence) and ^1^H, ^13^C HMBC (heteronuclear multiple bond correlation) were employed to establish one- and multiple-bond ^1^H–^13^C connectivities, respectively. Transfer delay for the long-range ^1^H, ^13^C correlations was set to 62 ms, based on ^n^J_C,H_ couplings of 8 Hz. 2D ^1^H,^13^C HSQC–TOCSY experiment (*t*_m_ = 90 ms) was utilized to lift degeneracy in ^1^H chemical shifts when necessary. 2D ROESY (rotating frame Overhauser effect spectroscopy) with a spinlock time of 200 ms was employed to confirm long-range correlations in ^13^C-HMBC spectrum and establish through-space connectivities between protons. ^1^H spectrum of hydrolyzed MAAs in 2 M D_2_SO_4_ was measured using 30-degree flip angle and recycle delay of 14 seconds.

### Whole genome sequencing of *Nostoc* sp. UHCC 0302

A four-week-old 40 ml axenic culture of *Nostoc* sp. UHCC 0302 was harvested by centrifuging at 7000 g for 5 minutes to extract genomic DNA, using a standard phenol–chloroform and ethanol precipitation method. Extracted DNA was dissolved in 30 μl of 5 mM trisHCl at pH 8. DNA quantity and quality were assessed using Nanodrop 1 spectrophotometer (Thermo Fisher Scientific).

Pacbio Sequel II instrument was used for the sequencing reactions and the initial assemblies were done according to the Pacbio's SMRTlink version 9 microbial assembly user guide at the University of Helsinki sequencing centre. *De novo* genome assemblies were obtained with Flye v2.9.^44^ The assembled scaffolds were classified with Kaiju v1.7.2^45^ at the phylum level and separated using in-house scripts to obtain only cyanobacterial scaffolds. The circularity of sequences was checked with Bandage v0.8.1^46^ and the completeness and contamination of the genomes were assessed with CheckM v1.0.13.^47^

### Reconstruction and heterologous expression of the MAA biosynthetic gene cluster of *Nostoc* sp. UHCC 0302 in *E. coli* BL21 (DE3)

For our preliminary analysis we cloned the *mysABCJ*_*1*_*D*_*1*_*HG*_*1*_ into the *NcoI* and *NheI* sites of the pET28a+ plasmid vector with T7/lac promotor and a kanamycin resistance cassette (Kn) to check whether the cyanobacterial MAA biosynthetic enzymes could produce MAAs in *E. coli* BL21 (DE3). We first amplified the *mysABCJ*_*1*_*D*_*1*_ fragment from the genomic DNA of *Nostoc* sp. UHCC 0302 using the polymerase chain reaction (PCR). Primer pair P1 annealing to *mysA*, was strategically engineered with *NcoI* restriction site and custom ribosomal binding sequence (RBS) optimized (Tables S9 and S10, ESI†) for maximal translation efficiency as per *De Novo* DNA RBS calculator v2.2.^48^ To complement this, reverse primer P2, designed to bind to *mysD*, incorporated an additional *BamHI* site (Table S9, ESI†). The PCR reaction was executed utilizing Q5 polymerase (NEB) following the manufacturer's protocol. Amplified DNA fragments were cleaned with NucleoSpin Mini PCR clean-up kit (Macherey-Nagel, Finland) and then digested using the FastDigest NcoI and BamHI enzymes (Thermo Scientific™, Thermofisher). In parallel we also digested and dephosphorylated the pET28a+ plasmid vector using the same restriction enzyme pair and the FastAP enzyme (Thermo Scientific™, Thermofisher) for conventional cloning of the fragment using a T4 DNA Ligase (Thermo Scientific™, Thermofisher). This construct was propagated in chemically competent *E. coli* (DH5α) using a standard transformation and culturing protocol. The plasmid DNA was then harvested using the PureLink™ Quick Plasmid Miniprep Kit (Intvitrogen™, Thermofisher Scientific) and confirmed the construct sequence with Next Generation Sequencing (NGS) services provided by Eurofins Genomics (Germany) using the S1 and S2 sequencing primers. Similarly, we also generated the constructs *mysABC* and *mysABCJ*_*1*_ with the primer pairs P1–P3 and P1–P4 respectively. In parallel, we have also amplified the *mysHG*_*1*_ using the primer pair P5 and P6 with 20–25 bp long homologous regions to the *mysD*_*1*_ enzyme and the downstream of the *SalI* site in the pET28a+ vector respectively. This then allowed us to insert the *mysHG*_*1*_ in the same orientation into the *mysABCJ*_*1*_*D*_*1*_ in pET28a+ construct using the NEBuilder® HiFi DNA Assembly Master Mix (New England BioLabs) to obtain the full cluster in the expression system as *mysABCJ*_*1*_*D*_*1*_*HG*_*1*_::pET28a+. Similar approach was taken to also generate the construct *mysABCJ*_*1*_*D*_*1*_*H*, where reverse primer P7 annealing to *mysH* with pET28a+ overlapping sequence was used along with P5 (Table S7, ESI†). The same propagation and validation protocol was followed as before and both constructs were then transformed into chemically competent *E. coli* BL21 (DE3) using the standard chemical transformation protocol.

To confirm whether the transformed *E. coli* could produce the MAA structural variants anticipated we set up a small-scale induced expression culture experiment. The *E. coli* BL21 (DE3) colonies were grown in 5 ml Luria Bertani (LB) broth overnight at 37 °C degrees with constant shaking at 185 rpm (shaker) to be re-inoculated to a fresh 5 ml LB medium and induced with 0.1 mM IPTG the next day. After the induction the cultures were placed in 18 °C with constant shaking at 185 rpm for another 24 hours. Cultures were then harvested by centrifuging at 8000 g using Eppendorf centrifuge 5804 R (Germany) and cell pellets were collected for lyophilisation and methanol extraction as described before. The extract samples were then analysed with HR-LCMS.

### Higher MAA production in recombinant *E. coli* BL21 (DE3) for purification and characterization of hexosyl-palythine-Thr

To achieve large-scale production of hexosyl-palythine-Thr for further analysis, we employed the following procedure which allowed us maximum production in *E. coli.* Overnight cultures were established by inoculating 40 ml of LB + 50 μg ml^−1^ Kn with *E. coli* BL21 (DE3)::*mysABCJ*_1_*D*_1_*HG*_1_::pET28a+, following the same protocol as previously described. Subsequently, these cultures were used to inoculate eight 2 L glass flasks containing 500 ml of LB + 50 μg ml^−1^ Kn and 1% Xylose. The flasks were then placed in a MaxQ™ 8000 shaking incubator (Thermo Scientific™) at 37 °C with constant shaking at 200 rpm until reaching an approximate OD_600_ value of 0.5 AU (approximately 2 hours). Afterward, the cultures were allowed to cool for 15 minutes at 4 °C before being induced with 0.1 mM IPTG. Following induction, the flasks were returned to the shaking incubator and maintained at 18 °C with shaking at 200 rpm for 24 hours. The cultures were subsequently harvested for the extraction and purification of 450 Da hexosyl-palythine-Thr as before with the cyanobacterial cultures. Based on the estimated calculations we did using the previously purified MAAs as standards, our recombinant *E. coli* clone was able to produce 10× more MAAs than *Nostoc* sp. UHCC 0302 in only 24 hours (Table S19, ESI†).

### Synthetic constructs to test MysD_1_*vs.* MysD_2_ activity in MAA biosynthesis.

To assess the functionality of the enzymes encoded in the distant and partial *mysD*_*2*_*J*_*2*_*G*_*2*_ gene cluster of *Nostoc* sp. UHCC 0302 we have designed and ordered a codon optimized expression system with custom calculated RBS (Table S8, ESI†) from GenScript Biotech Corporation (Netherlands, EU). *mysABCD*_*1*_*D*_*2*_*H* was cloned into *NheI* and *NcoI* sites in pBAD/HIS-B plasmid vector at the that harbors an ampicillin resistance gene and the araBAD system which allows for tighter regulation of the expression. We modified *mysABCD*_*1*_*D*_*2*_*H* in pBAD/HIS-B using the added restriction sites *SalI* and *SacI* to also obtain the constructs *mysABCD*_*1*_, and *mysABCD*_*2*_ in pBAD/HIS-B respectively. All reactions were done using FastDigest version of the enzymes from Thermo Scientific™, Thermofisher. We confirmed these new constructs *via* PCR reactions using the X-primer series (Table S7, ESI†) and 0.8% agarose gel electrophoresis. These clones were then cultured and induced in standard conditions suggested for the araBAD system and MAAs produced were analysed HR-LCMS as described before (Table S20, ESI†).

### Bioinformatic analyses of the MGA-ligase and glycosyltransferase enzymes across cyanobacteria

The MAA biosynthetic gene cluster was examined within high-quality genomic assemblies for a comparative analysis with *Nostoc* sp. UHCC 0302. Specifically, the *mysABCD* genes were screened in a collection of 336 publicly available complete cyanobacterial genomes from Genbank database (as of October 30th, 2023) using blastp^29^ with a minimum alignment identity of 40% and coverage of 70%. The *mysABCD*_*1*_ amino acid sequences from *Nostoc* sp. UHCC 0302 served as the reference for this analysis. Subsequently, MysD and glycosyltransferases amino acid sequences were used to build maximum likelihood phylogenetic trees, inferred with IQ-TREE v1.7^49^ using default parameters, with the substitution models JTTDCMut + G4 for the MysD tree and JTTDCMut + F + I + G4 for the glycosyltransferases, automatically chosen by the software. The generated trees were visualized and edited in the iTOL v6 program and Inkscape (0.92.4).^50,51^ SWISS-Model homology-based protein model analyses were done using protein sequences of MysJ_1/2_ and MysG_1/2_.^52^

## Author contributions

D. F. and S. A. conceptualized and designed the study. S. A. conducted the experiments and wrote the manuscript. M. P. aided in the design and implementation of the heterologous expression systems. E.D. performed whole genome assembly and constructed the phylogenetic trees. M. W. assisted with HR-LCMS analyses and aided in the purification of compounds. J. J. and P. P. carried out NMR analyses and contributed to the structural characterization of compounds. All authors reviewed and approved the final manuscript.

## Data availability

The complete genome sequence data for *Nostoc* sp. UHCC 0302 generated for this article is available at GenBank sequence repository under the assession number CP151099. Additional data supporting this article including HR-LCMS chromatograms of the MAAs from additional and control constructs, NMR data, genome, primers used, and the MysJ_1/2_ and MysG_1/2_ phylogenetic trees are provided in the ESI.†

## Conflicts of interest

There are no conflicts of interest.

## Supplementary Material

CB-OLF-D4CB00128A-s001
