# Supplementary material for: A refactored biosynthetic pathway for the production of glycosylated microbial sunscreens
Source: RSC Chem Biol. 2024 Aug 20;5(10):1035–44. doi: 10.1039/d4cb00128a (PMC11378024; doi:10.1039/d4cb00128a)
Supplement: CB-005-D4CB00128A-s001 [file CB-005-D4CB00128A-s001.pdf]

Supplementary Information (SI) for RSC Chemical Biology.
This journal is © The Royal Society of Chemistry 2024

**A refactored biosynthetic pathway for the production of glycosylated microbial sunscreens**

Sıla Arsın[1], Maija Pollari[1,3], Endrews Delbaje[2], Jouni Jokela[1], Matti Wahlsten[1], Perttu Permi[4, 5], David Fewer[1]

[1] Department of Microbiology, Faculty of Agriculture and Forestry, University of Helsinki, 00014 Helsinki, Finland.

[2] Faculdade de Ciências Farmacêuticas de Ribeirão Preto, Universidade de São Paulo, Ribeirão Preto, Brazil

[3] Department of Agricultural Sciences, Faculty of Agriculture and Forestry, University of Helsinki, 00014 Helsinki, Finland.

[4]Department of Chemistry, University of Jyväskylä, 40014 Jyväskylä, Finland

[5]Department of Biological and Environmental Science, Nanoscience Center, University of Jyväskylä, 40014 Jyväskylä, Finland

*Corresponding author

E-mail address: david.fewer@helsinki.fi

## Supplementary Information

## Table of Contents

**Table S1.** MAAs variants detected by HR-LCMS from the *Nostoc* sp. UHCC 0302 extract....................S2

**Figure S1.** MS-ES$^+$ chromatograms of the detected MAA variants and intermediates....................S2

**Table S2.** Product ions of 568 Da MAA from MS$^E$ spectrum ....................S3

**Table S3.** NMR data of 568 Da MAA in $D_2O$ ....................S4

**Figure S2** Product ion spectrum of protonated 568 Da MAA ....................S5

**Figure S3.** Partly annotated proton spectrum of 568 Da MAA in $D_2O$. ....................S6

**Figure S4.** Annotated $^1H$-$^1H$ DQF-COSY spectrum of 568 Da MAA in $D_2O$ ....................S7

**Figure S5.** $^1H$-$^1H$ TOCSY (90 ms) spectrum of 568 Da MAA in D2O. ....................S8

**Figure S6.** Annotated $^{13}C$-HSQC-TOCSY spectrum of 568 Da MAA in $D_2O$....................S9

**Figure S7.** Annotated edited $^1H$-$^{13}C$ HSQC spectrum of 568 Da MAA in $D_2O$. ....................S9

**Figure S8.** Annotated $^1H$-$^{13}C$ HMBC spectrum of 568 Da MAA in $D_2O$ ....................S10

**Figure S9.** HMBC and COSY correlations of 568 Da MAA, and its absorbtion maxima at 323 nm ....................S11

**Figure S10.** Partial $^1H$ spectra of 568 Da MAA acid hydrolysate in 2 M $D_2SO_4$ (in $D_2O$) ....................S11

**Figure S11.** Ion coding marked to the 450 Da MAA structure (A). ....................S12

**Table S5.** Product ions of 450 Da MAA from MS$^E$ (E: elevated collision energy) spectrum. ....................S13

**Table S6.** NMR data of 450 Da MAA in $D_2O$ ....................S14

**Figure S12.** Annotated proton spectrum of 450 Da MAA in $D_2O$....................S14

**Figure S13.** MS$^E$ (E: elevated collision energy) of protonated 450 Da MAA ....................S15

**Figure S14.** Annotated $^1H$-$^1H$ DQF-COSY spectrum of 450 Da MAA in $D_2O$....................S15

**Figure S15.** Annotated $^1H$-$^1H$ ROESY spectrum of 450 Da MAA in $D_2O$. ....................S16

**Figure S16.** Annotated edited $^1H$-$^{13}C$ HSQC spectrum of 450 Da MAA in $D_2O$ ....................S16

**Figure S17.** Annotated $^1H$-$^{13}C$ HMBC spectrum of 450 Da MAA in $D_2O$....................S17

**Table S7.** Complete genome assembly stats for *Nostoc* sp. UHCC 0302....................S17

**Table S8.** MAA biosynthetic enzymes of *Nostoc* sp. UHCC 0302 as annotated and deposited in GenBank ....................S18

**Table S9.** Primers used for the cloning of MAA biosynthetic gene clusters of *Nostoc* sp. UHCC 0302 ....................S18

**Table S10.** Optimal RBS sequences calculated for each MAA biosynthetic gene cluster enzyme of *Nostoc* sp. UHCC 0302 ........................................................................................................................................S19

**Figure S18.** HR-LCMS chromatograms of different MAA variants detected from the extracts of recombinant *Escherichia coli* BL21 (DE3)::pET28a+ constructs ..................................................................................S19

**Figure S19.** Total MAA production comparison between *Nostoc* sp. UHCC 0302 and *Escherichia coli* BL21 (DE3)::pET28a+ constructs ....................................................................................................................S20

**Figure S20.** HR-LCMS chromatograms of different MAA variants detected from the extracts of recombinant *Escherichia coli* BL21 (DE3)::pBAD/HisB constructs ........................................................................................S21

**Figure S21.** Phylogenetic distribution of the $MysJ_{1/2}$ and $MysG_{1/2}$ across complete cyanobacterial genomes .......S22

**Table S11.** Homology based protein structure prediction summary using Swiss-Model for *Nostoc* sp. UHCC 0302 $MysJ_{1/2}$ and $MysG_{1/2}$ enzymes……………………………………………………...…………………………S23

**Table S1.** MAA intermediates and variants detected from *Nostoc* sp. UHCC 0302 by HR-LCMS based on their UV absorbtion at 320 nm and their abundance is based on the area of absorbtion in percentages (Area %). Retention time: RT, (experimental) *m/z* in [M+ H] $^{+}$. (Hexose: Hex, Pentose: Pen, Galactose: Gal, Glucose: Glu, Xylose: Xyl)

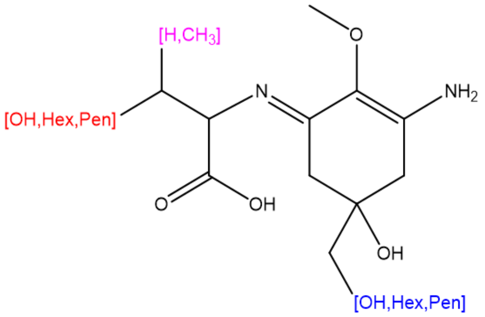


| # | MAA | RT (min) | *m/z* | Area (320nm) | Area (%) |
|---|---|---|---|---|---|
| 1 | Thr-4DG-$NH_2$- Xyl | 6.40 | 421.18 | 197 | 6.4 |
| 2 | Xyl-Thr-4DG-$NH_2$ | 6.61 | 421.18 | 32 | 1.1 |
| 3 | Ser-4DG-$NH_2$-Xyl | 6.82 | 407.17 | 377 | 12.3 |
| 4 | Thr-4DG-$NH_2$-Glc | 7.10 | 451.19 | 549 | 17.8 |
| 5 | Gal-Thr-4DG-$NH_2$- Xyl | 7.79 | 583.24 | 320 | 10.4 |
| 6 | Gal-Ser-4DG-$NH_2$- Xyl | 8.22 | 569.22 | 1602 | 52.1 |

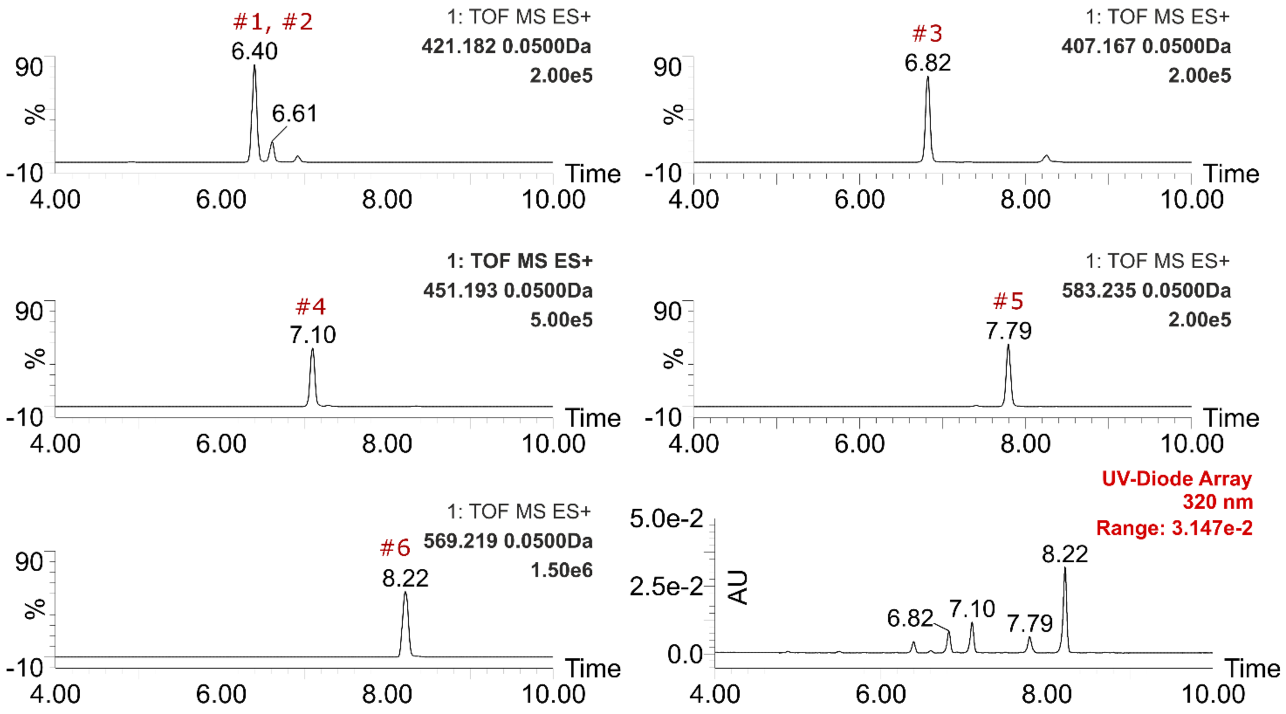


**Figure S1.** MS-ES$^{+}$ and UV (320 nm) chromatograms showing the detected MAA intermediates and variants from the *Nostoc* sp. UHCC 0302 methanol extracts.

**Table S2.** Product ions of 568 Da MAA from $MS^E$ spectrum. Codes are marked to the 568 Da MAA molecule in Figure S2. Δ = difference of calculated (Calc) and experimental (Exp) ion masses in parts per million (ppm).

| Product ion | | | *m/z* | | |
|---|---|---|---|---|---|
| **code** | | **Neutral loss** | **Calc** | **Exp** | **Δ (ppm)** |
| | $[M+K]^+$ | | 607.1747 | 607.1732 | -2.5 |
| | $[M+Na]^+$ | | 591.2008 | 591.1999 | -1.6 |
| | $[M+H]^+$ | | 569.2188 | 569.2195 | 1.1 |
| a | $[M+H]^+$ | $CH_3^{\bullet}$ | 554.1954 | 554.1943 | -2.0 |
| b | $[M+H]^+$ | $CH_2O$ | 539.2083 | 539.2081 | -0.4 |
| b | $[M+H]^+$ | $CH_3OH$ | 537.1926 | 537.1922 | -0.9 |
| c | $[M+H]^+$ | $C_2H_2N^{\bullet}$ | 529.2001 | 529.2005 | 0.6 |
| d | $[M+H]^+$ | $CO_2$ | 525.2290 | 525.2291 | 0.1 |
| d | $[M+H]^+$ | HCOOH | 523.2134 | 523.2139 | 0.9 |
| e | $[M+H]^+$ | $C_4H_8O_4$ | 449.1766 | 449.1751 | -3.4 |
| f | $[M+H]^+$ | Xyl | 437.1766 | 437.1759 | -1.7 |
| g | $[M+H]^+$ | Gal | 407.1660 | 407.1659 | -0.4 |
| a-g | $[M+H]^+$ | Gal, $CH_3^{\bullet}$ | 392.1426 | 392.1420 | -1.5 |
| h | $[M+H]^+$ | Gal, $H_2O$ | 389.1555 | 389.1542 | -3.4 |
| b-g | $[M+H]^+$ | Gal, $CH_2O$ | 377.1555 | 377.1546 | -2.4 |
| d-g | $[M+H]^+$ | Gal, HCOOH | 361.1605 | 361.1594 | -3.3 |
| d-h | $[M+H]^+$ | Gal, $CO_2$, $H_2O$ | 345.1656 | 345.1649 | -2.3 |
| b-d-g | $[M+H]^+$ | Gal, HCOOH, $CH_2O$ | 331.1500 | 331.1490 | -3.1 |
| i | $[M+H]^+$ | $GalOC_3H_3O_2$ | 319.1500 | 319.1487 | -4.2 |
| i | $[M+H]^+$ | $GalOC_3H_5O_2$ | 317.1343 | 317.1337 | -2.1 |
| b-i | $[M+H]^+$ | $GalOC_3H_3O_2$, $CH_3^{\bullet}$ | 304.1265 | 304.1259 | -2.1 |
| f-g | $[M+H]^+$ | Gal, Xyl | 275.1238 | 275.1239 | 0.3 |
| a-f-g | $[M+H]^+$ | Gal, Xyl, $CH_3^{\bullet}$ | 260.1003 | 260.1001 | -0.9 |
| f-h | $[M+H]^+$ | Gal, Xyl, $H_2O$ | 257.1132 | 257.1131 | -0.6 |
| b-f-g | $[M+H]^+$ | Gal, Xyl, $CH_2O$ | 245.1132 | 245.1130 | -1.0 |
| b-f-g | $[M+H]^+$ | Gal, Xyl, $CH_3OH$, $2xH_2O$ | 207.0764 | 207.0768 | 1.6 |
| b-f-j | $[M+H]^+$ | GalOCH, Xyl, $CH_2O$, $H_2O$ | 197.0921 | 197.0918 | -1.6 |
| f-i | $[M+H]^+$ | $GalOC_3H_3O_2$, Xyl, 2H | 185.0921 | 185.0921 | -0.1 |
| a-f-i | $[M+H]^+$ | $GalOC_3H_3O_2$, Xyl, $CH3^{\bullet}$ | 172.0842 | 172.0845 | 1.2 |
| | $[M+H]^+$ | $C_{13}H_{28}O_{14}$ | 161.0709 | 161.0714 | 2.5 |

**Table S3.** NMR data of 568 Da MAA in $D_2O$.

| No | $\delta_H$ | mult., *J*(Hz) | $\delta_C$ | HMBC |
|---|---|---|---|---|
| **Palythine-serine** | | | | |
| 1 | - | | 160.9 | |
| 2 | - | | 125.1 | |
| 3 | - | | 160.9 | |
| 4* | 2.80 | | 34.1 | 2, 3, 5, 6 |
| 5 | - | | 70.6 | |
| 6* | 2.70 | | 36.0 | 1, 2, 4, 5, 7 |
| 7a | 3.43 | d, 10.0 | 73.3 | 5, 6, 1' |
| 7b | 3.72 | d, 10.1 | | 5, 6, 1' |
| 8 | 3.64 | s | 59.4 | 2 |
| 9 | 4.44 | dd, 6.2, 3.6 | 59.0 | 3, 10, 11 |
| 10 | - | | 174.3 | |
| 11a | 4.03 | dd, 11.0, 3.5 | 70.3 | 9, 10, 1" |
| 11b | 4.29 | dd, 11.0, 6.2 | | 9, 10, 1" |
| **α-D-xylopyranose** | | | | |
| 1' | 4.89 | d, 3.7 | 98.9 | 5', 7 |
| 2' | 3.54 | | 71.7 | 3' |
| 3' | 3.65 | | 73.3 | 2', 4' |
| 4' | 3.60 | | 69.6 | 3', 5' |
| 5a' | 3.45 | | 61.6 | 1', 3', 4' |
| 5b' | 3.70 | | | 1', 3', 4' |
| **β-D-galactopyranose** | | | | |
| 1" | 4.37 | d, 8.0 | 103.2 | 2", 3", 5", 11 |
| 2" | 3.49 | | 70.9 | 1", 3" |
| 3" | 3.61 | | 72.9 | 2" |
| 4" | 3.87 | dd, 0.8, 3.3 | 68.9 | 2", 3" |
| 5" | 3.64 | | 75.5 | 1", 4", 6" |
| 6a" | 3.69 | | 61.3 | 4", 5" |
| 6b" | 3.74 | | | 4", 5" |

* = 4 and 6 signals can be interchanged.

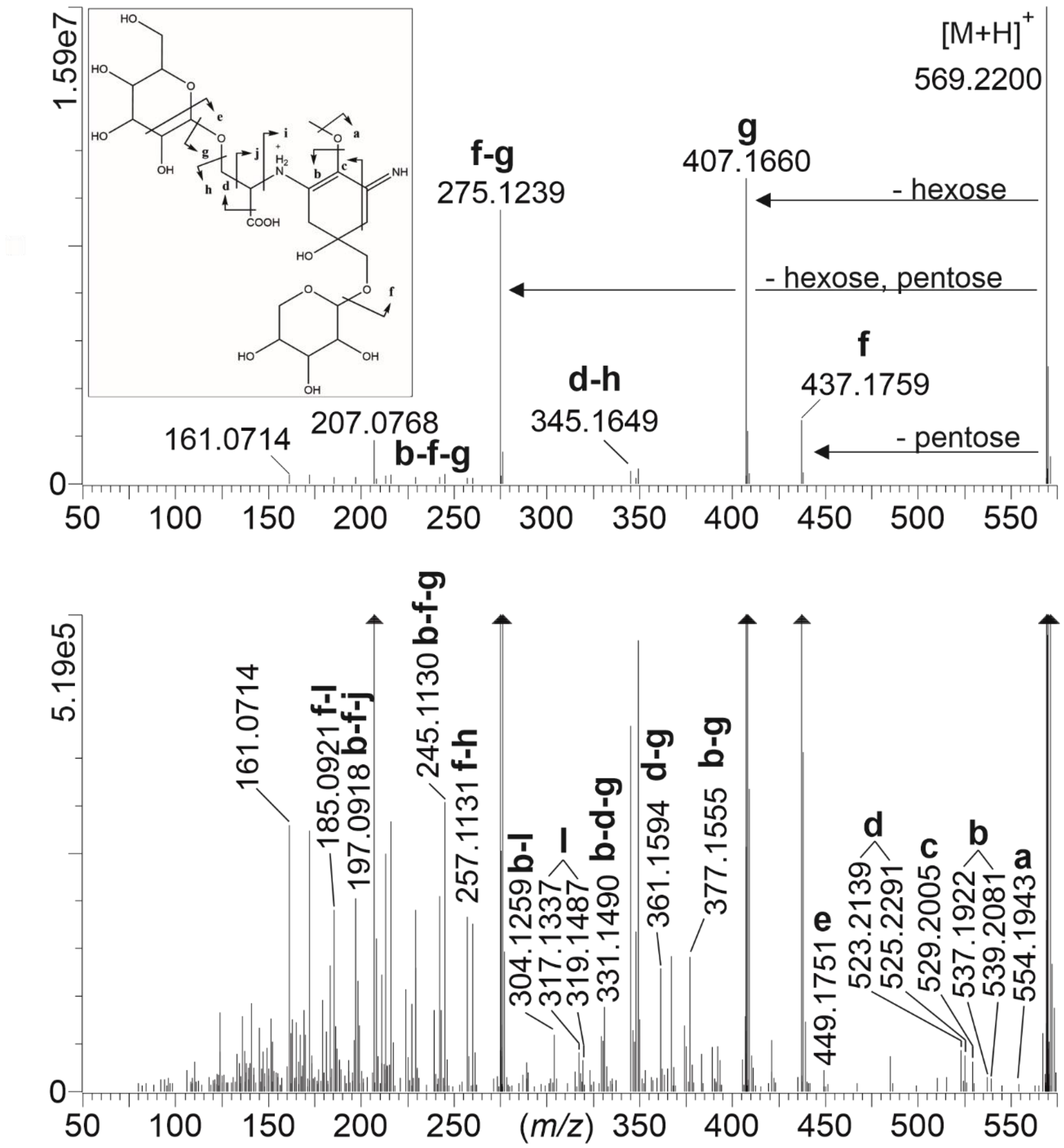


**Figure S2.** Product ion spectrum of protonated 568 Da MAA. The lower panel shows the low intensity ions.

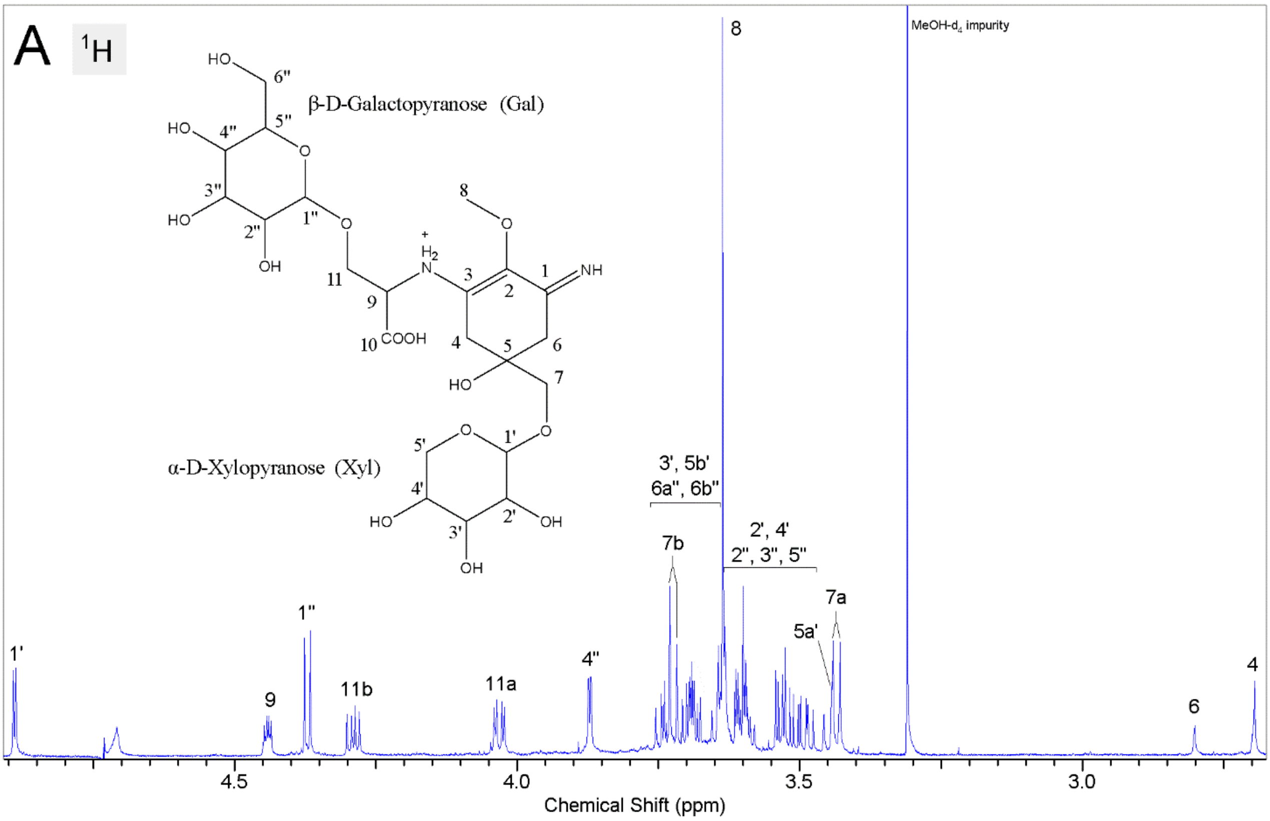


**Figure S3.** Partly annotated proton spectrum of 568 Da MAA in $D_2O$.

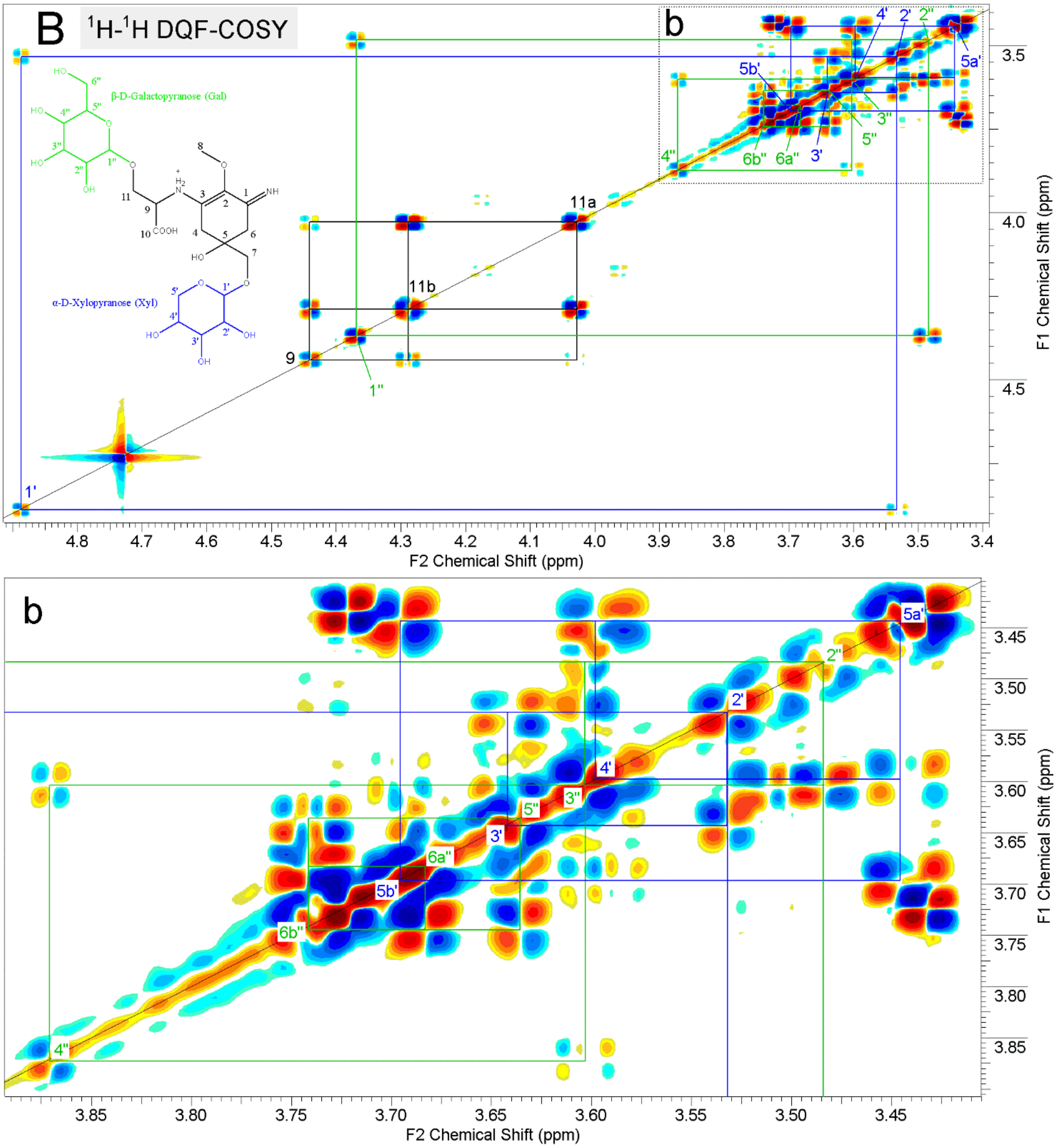


**Figure S4.** Annotated $^1$H-$^1$H DQF-COSY spectrum of 568 Da MAA in $D_2O$ with enlargement from area b.

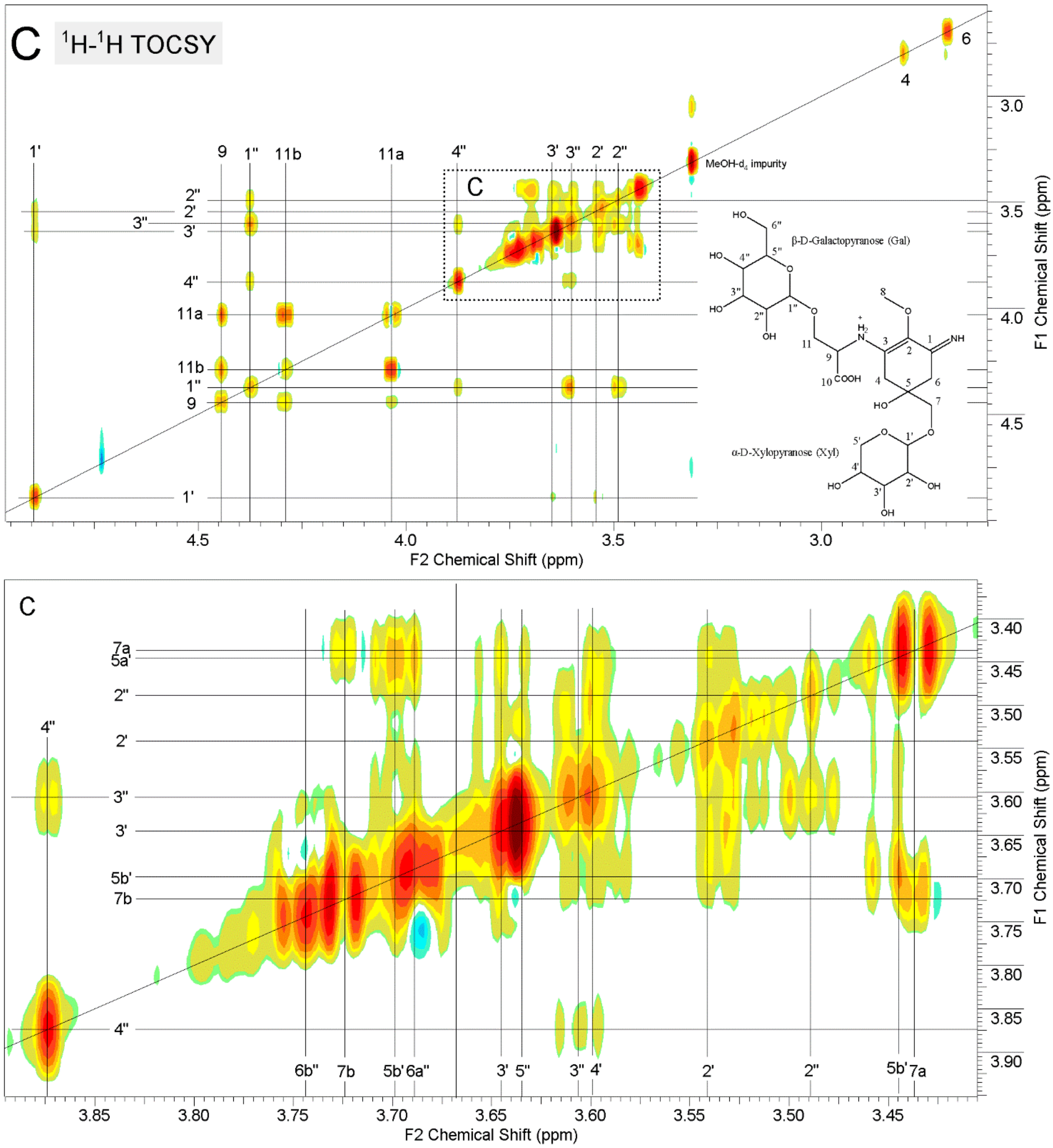


**Figure S5.** $^{1}H$-$^{1}H$ TOCSY (90 ms) spectrum of 568 Da MAA in $D_2O$.

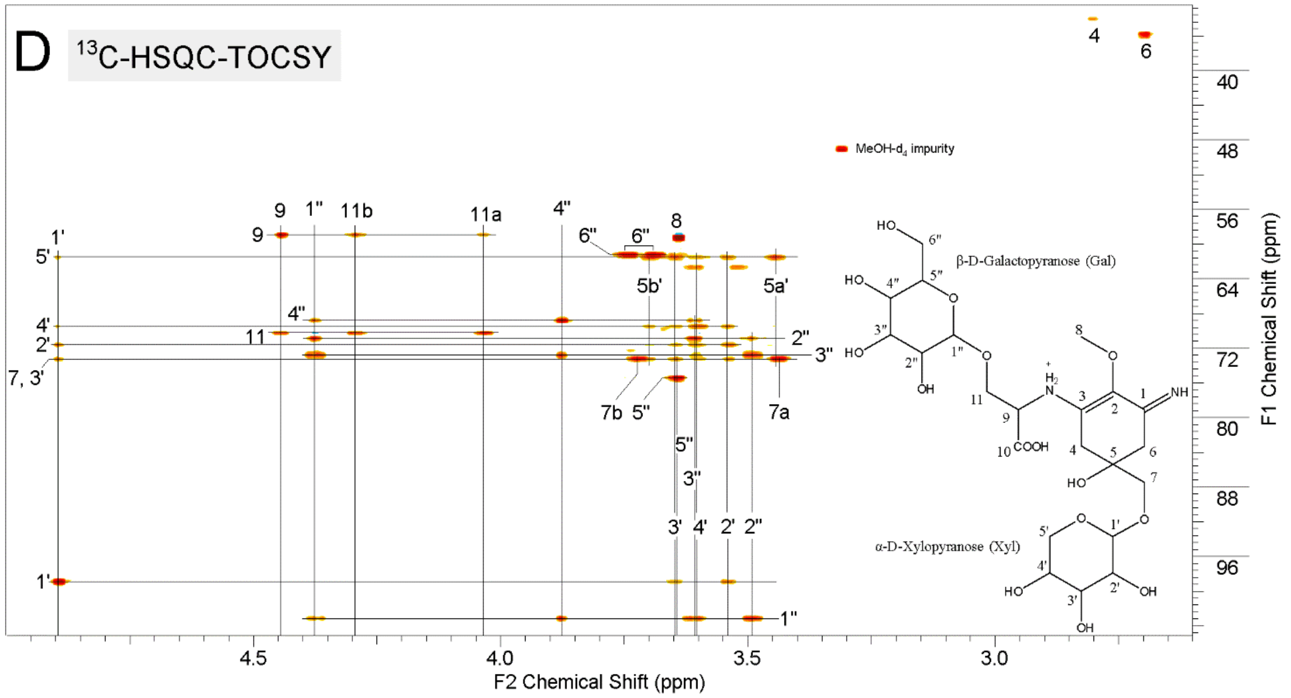


**Figure S6.** Annotated $^{13}C$-HSQC-TOCSY spectrum of 568 Da MAA in $D_2O$.

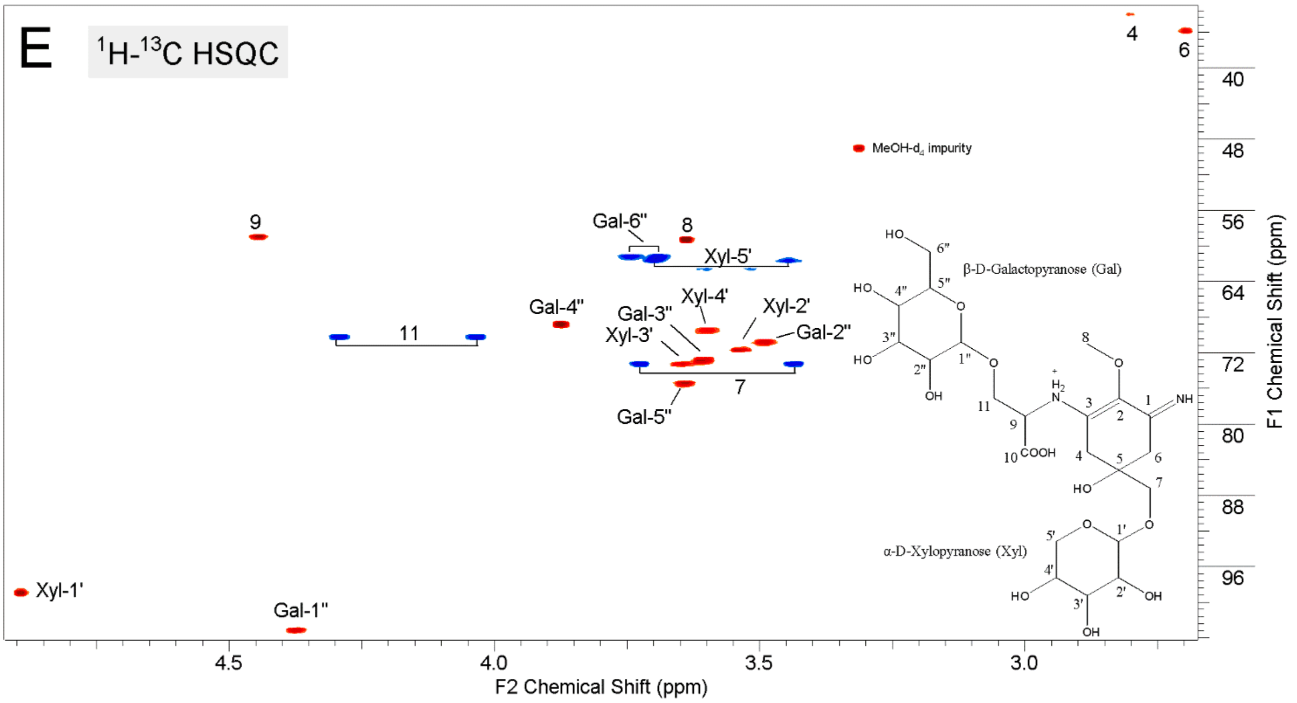


**Figure S7.** Annotated edited $^{1}H$-$^{13}C$ HSQC spectrum of 568 Da MAA in $D_2O$.

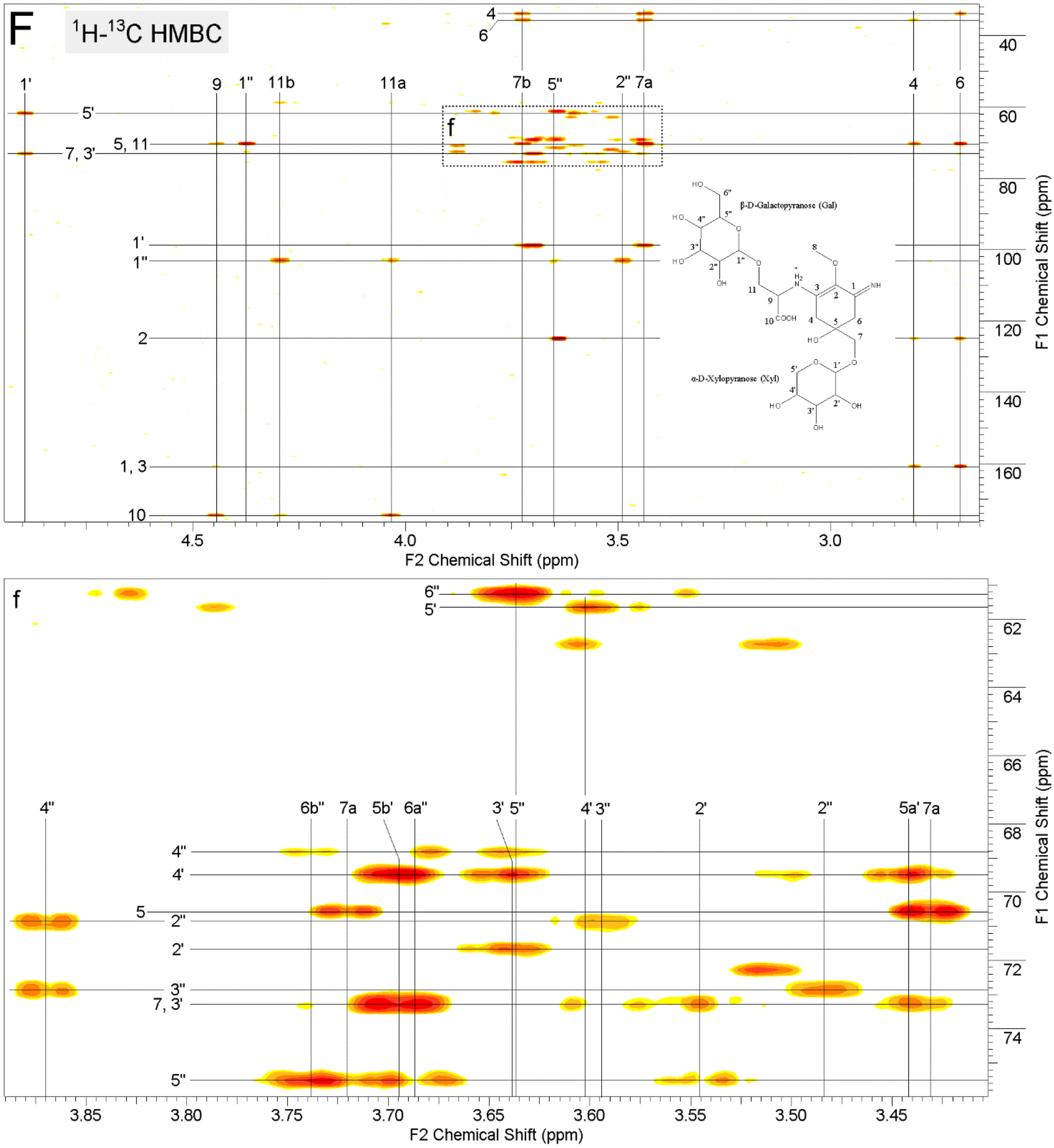


**Figure S8.** Annotated $^1$H-$^{13}$C HMBC spectrum of 568 Da MAA in $D_2O$ with enlargement from area f.

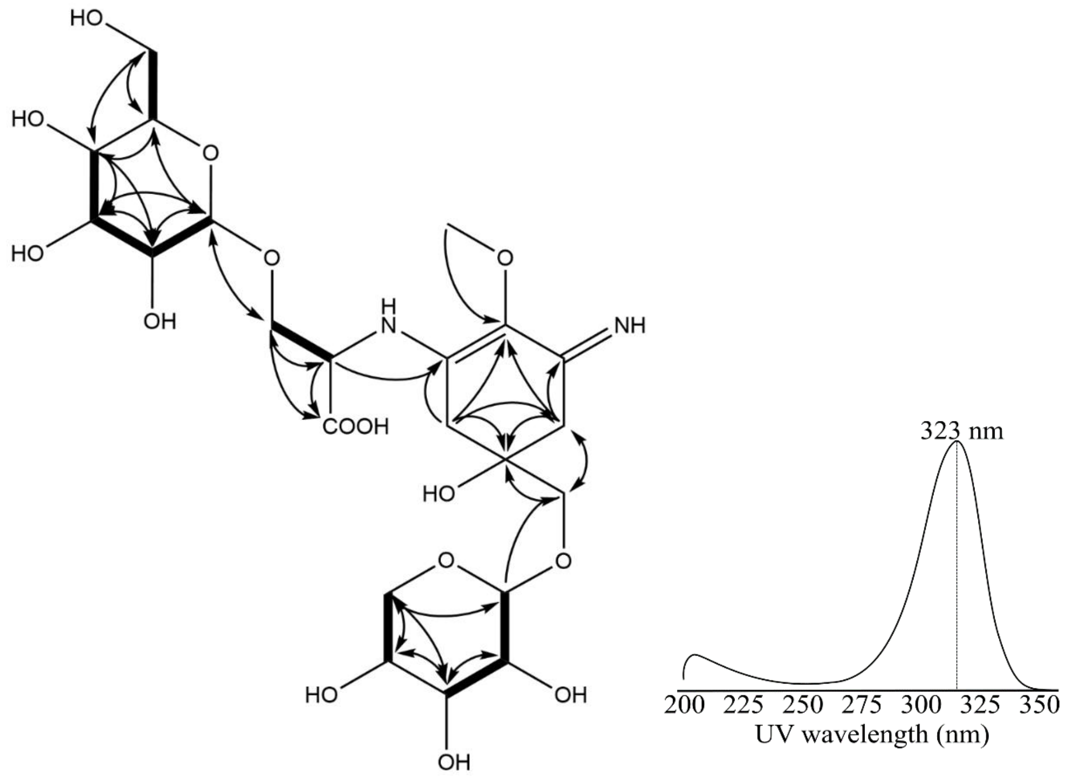


**Figure S9.** HMBC and COSY correlations of 568 Da MAA, and its absorbtion maxima at 323 nm.

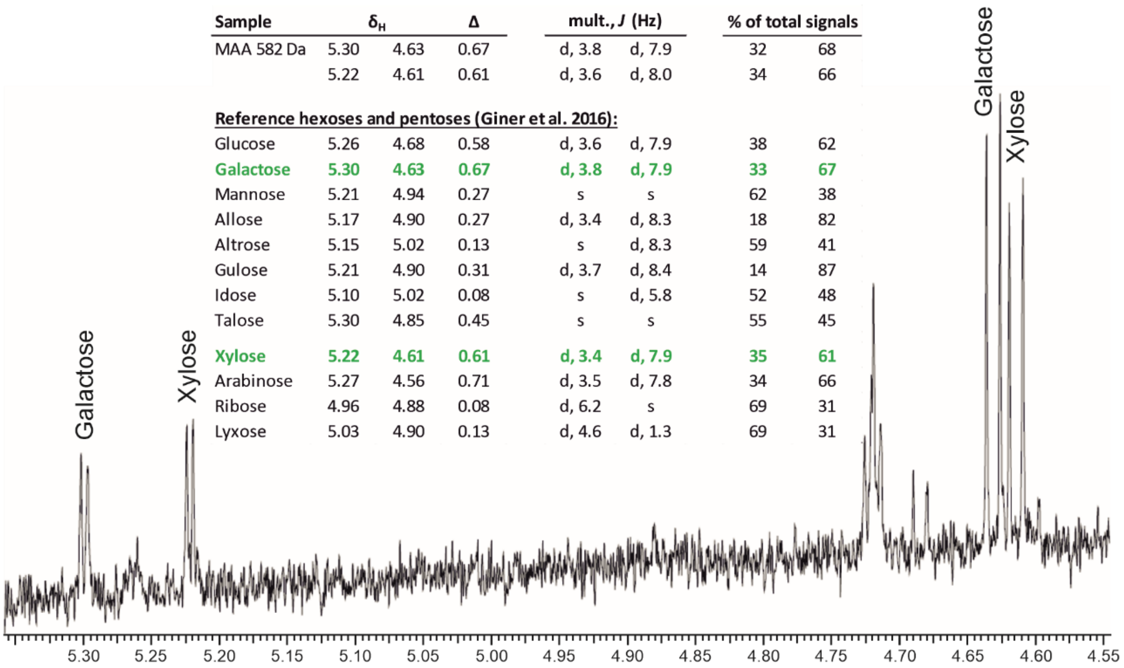


| Sample | $\delta_H$ | | Δ | mult., *J* (Hz) | | % of total signals | |
|---|---|---|---|---|---|---|---|
| MAA 582 Da | 5.30 | 4.63 | 0.67 | d, 3.8 | d, 7.9 | 32 | 68 |
| | 5.22 | 4.61 | 0.61 | d, 3.6 | d, 8.0 | 34 | 66 |
| Reference hexoses and pentoses (Giner et al. 2016): | | | | | | | |
| Glucose | 5.26 | 4.68 | 0.58 | d, 3.6 | d, 7.9 | 38 | 62 |
| Galactose | 5.30 | 4.63 | 0.67 | d, 3.8 | d, 7.9 | 33 | 67 |
| Mannose | 5.21 | 4.94 | 0.27 | s | s | 62 | 38 |
| Allose | 5.17 | 4.90 | 0.27 | d, 3.4 | d, 8.3 | 18 | 82 |
| Altrose | 5.15 | 5.02 | 0.13 | s | d, 8.3 | 59 | 41 |
| Gulose | 5.21 | 4.90 | 0.31 | d, 3.7 | d, 8.4 | 14 | 87 |
| Idose | 5.10 | 5.02 | 0.08 | s | d, 5.8 | 52 | 48 |
| Talose | 5.30 | 4.85 | 0.45 | s | s | 55 | 45 |
| Xylose | 5.22 | 4.61 | 0.61 | d, 3.4 | d, 7.9 | 35 | 61 |
| Arabinose | 5.27 | 4.56 | 0.71 | d, 3.5 | d, 7.8 | 34 | 66 |
| Ribose | 4.96 | 4.88 | 0.08 | d, 6.2 | s | 69 | 31 |
| Lyxose | 5.03 | 4.90 | 0.13 | d, 4.6 | d, 1.3 | 69 | 31 |

**Figure S10.** Partial $^{1}H$ spectra of 568 Da MAA acid hydrolysate in 2 M $D_2SO_4$ (in $D_2O$) showing the match of anomeric protons signals to reference compounds presented in the table which values are from Giner et al., J Nat Prod 2016, 79, 2413-2417.

**Structural characterization of 450 Da hexose-palythine-threonine purified from *E. coli* BL21 (DE3) expressing the *mysABCJ₁D₁HG₁*::pET28a+**

High resolution QTOF-MS gave elemental composition of $C_{18}H_{30}N_2O_{11}$ (*m/z* 451.1919 for $[M+H]^+$, $\Delta - 0.7$ ppm) for the neutral 450 Da MAA compound. Fragmentation of the protonated molecule (Fig. S11, Table S5), UV maximum of 323 nm (Fig. S12) together with published MAA structural data indicate that the structure of 450 Da MAA is palythine-threonine (PT) with a hexose unit in 7-OH as presented in Figure S12. To get confirmation for the structure, 450 Da MAA was purified for NMR analysis. Sample was dissolved to $D_2O$ and $^1H$, $^1H$-$^1H$ DQF-COSY, edited $^1H$-$^{13}C$ HSQC and $^1H$-$^{13}C$ HMBC spectra were recorded. Numerical data is presented in Table S6 and spectra in Figures S13-17. All $\delta_H$ and $\delta_C$ signals and COSY/HMBC correlations for atom positions 1-12 were typical for palythine-threonine structure (Table S6). Also signals from a hexose (Hex) unit were recognized. HMBC correlation from PT H-7 and Hex anomeric C-1 was present meaning that Hex is connected to the PT unit 7-OH (Fig. S12). All $\delta_H$ and $\delta_C$ Hex signals and coupling constant $^3J_{H1,H2}$ of 3.7 Hz and $^1J_{H1,C1}$ of 170 Hz match with α-D-glycopyranose structure.

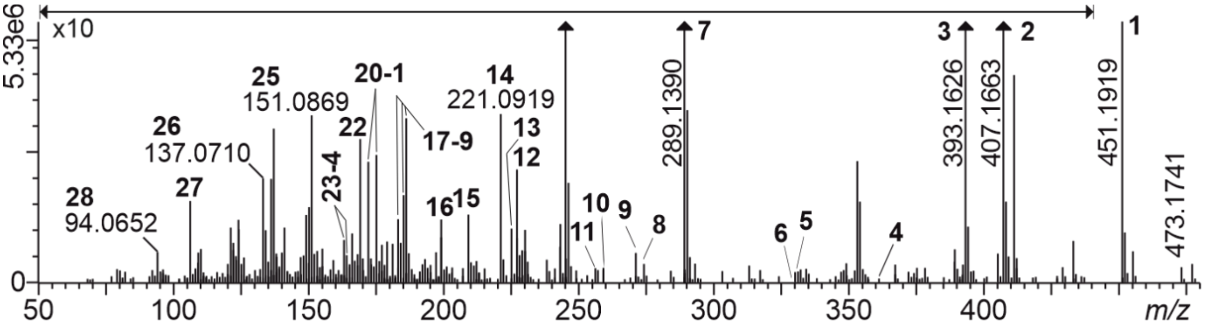


**Figure S11.** $MS^E$ (E: elevated collision energy) of protonated 450 Da MAA. Codes are marked to the 450 Da MAA molecule in Figure S11. The two headed horizontal arrow shows the 10 times magnified area.

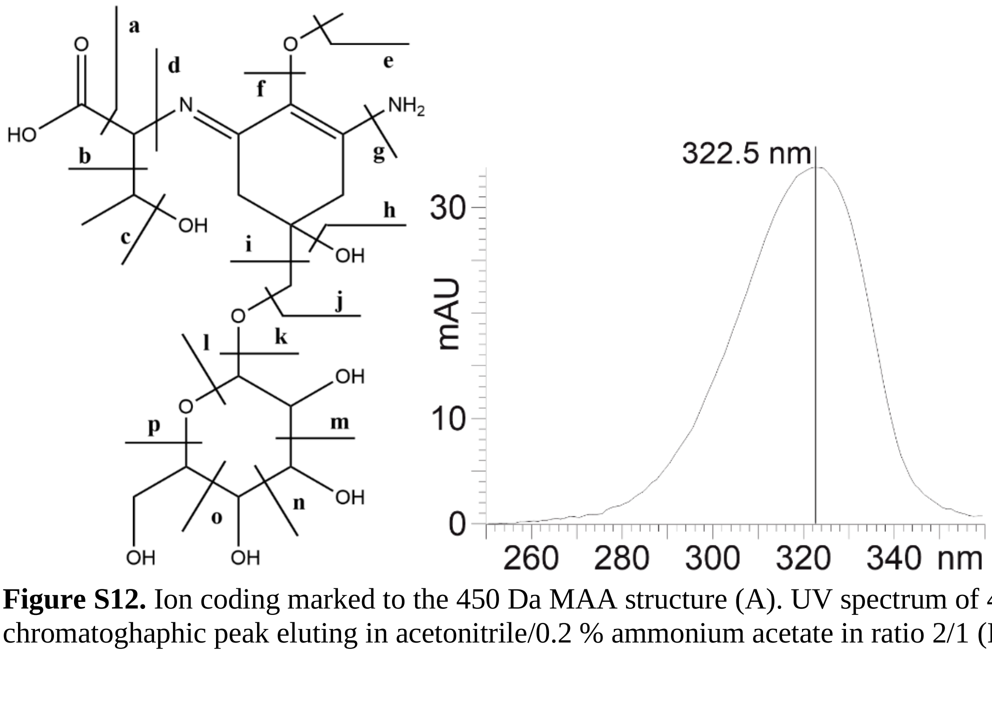


**Figure S12.** Ion coding marked to the 450 Da MAA structure (A). UV spectrum of 450 Da MAA from chromatoghaphic peak eluting in acetonitrile/0.2 % ammonium acetate in ratio 2/1 (B).

**Table S5.** Product ions of 450 Da MAA from $MS^E$ (E: elevated collision energy) spectrum. Codes are marked to the 450 Da MAA molecule in Figure S12. Δ = difference of calculated (Calc) and experimental (Exp) ion masses in parts per million (ppm).

| | Product ion | | *m/z* | | |
|---|---|---|---|---|---|
| **No** | **Code** | **Neutral loss** | **Calc** | **Exp** | **Δ (ppm)** |
| 1 | | - | 451.19223 | 451.1919 | -0.7 |
| 2 | o-p | $C_2H_4O$ | 407.16602 | 407.1663 | 0.6 |
| 3 | g-o-p | $C_2HO^•$, $NH_3$ | 393.16295 | 393.1626 | -1.0 |
| 4 | l-n | $C_3H_6O_3$ | 361.16054 | 361.1584 | -6.1 |
| 5 | l-m | $C_4H_8O_4$ | 331.14998 | 331.1486 | -4.3 |
| 6 | l-m | $C_4H_{10}O_4$ | 329.13433 | 329.1335 | -2.7 |
| 7 | k | Glc | 289.13941 | 289.1390 | -1.6 |
| 8 | e-k | Glc, $CH_3^•$ | 274.11594 | 274.1154 | -2.2 |
| 9 | j | Glc-OH | 271.12885 | 271.1284 | -1.8 |
| 10 | i | Glc-OCH | 259.12885 | 259.1282 | -2.7 |
| 11 | f-k | Glc-2H, $CH_3O^•$ | 256.10537 | 256.1042 | -4.8 |
| 12 | d-l-m | $C_4H_8O_3$, $C_4H_8O_4$ | 227.10263 | 227.1025 | -0.8 |
| 13 | d-l-m | $C_4H_8O_3$, $C_4H_{10}O_4$ | 225.08698 | 225.0867 | -1.5 |
| 14 | c-h-i | Glc-$OCH_3$, 2 x $H_2O$ | 221.09207 | 221.0919 | -1.0 |
| 15 | f-h-i | Glc-$OCH_3$, $CH_2O$, $H_2O$ | 209.09207 | 209.0918 | -1.5 |
| 16 | a-b-k | Glc, $C_3H_6O_3$ | 199.10772 | 199.1072 | -2.9 |
| 17 | d-k | Glc, $C_4H_7O_3^•$ | 186.09989 | 186.1001 | 0.9 |
| 18 | d-k | Glc, $C_4H_8O_3$ | 185.09207 | 185.0925 | 2.1 |
| 19 | d-k | Glc-2H, $C_4H_8O_3$ | 183.07642 | 183.0766 | 0.7 |
| 20 | a-c-h-i | Glc-$OCH_3$, HCOOH, 2 x $H_2O$ | 175.08659 | 175.0865 | -0.8 |
| 21 | d-e-k | Glc, $C_4H_8O_3$, $CH_3^•$ | 172.08424 | 172.0844 | 0.6 |
| 22 | d-j | Glc-OH, $C_4H_6O_3$ | 169.09715 | 169.0970 | -1.2 |
| 23 | a-f-h-i | Glc-$OCH_3$, $CO_2$, $CH_3O^•$, $H_2O$ | 164.09441 | 164.0944 | -0.4 |
| 24 | a-b-h-j | Glc-OH, $C_3H_6O_3$, $H_2O$ | 163.08659 | 163.0869 | 1.6 |
| 25 | d-h-j | Glc-OH, $C_4H_6O_3$, $H_2O$ | 151.08659 | 151.0869 | 1.7 |
| 26 | d-h-i | Glc-$OCH_3$, $C_4H_6O_3$, $H_2O$ | 137.07094 | 137.0710 | 0.1 |
| 27 | d-f-g-h-j | Glc-O, Thr, $CH_2O$, $H_2O$ | 106.06513 | 106.0651 | -0.8 |
| 28 | d-f-g-h-i | Glc-OCH, $C_4H_7NO_3$, $CH_2O$, $H_2O$ | 94.06513 | 94.0652 | 0.2 |

**Table S6.** NMR data of 450 Da MAA in $D_2O$. Chemical shift calibration with DSS.

| No | $\delta_H$ | mult., *J*(Hz) | $\delta_C$ | COSY | HMBC |
|---|---|---|---|---|---|
| **Core** | | | | | |
| 1 | - | | 163.5* | | - |
| 2 | - | | 127.5 | | - |
| 3 | - | | 163.3* | | - |
| 4a | 2.75* | 1.3, 17.1 | 38.7 | 6b | 2, 3, 5, 6, 7 |
| 4b | 3.04* | | | 6a | 2, 3, 5, 6, 7, 8 |
| 5 | - | | 73.2 | | - |
| 6a | 2.84* | 1.3, 17.5 | 36.7 | 4b | 1, 2, 4, 5, 7 |
| 6b | 3.06* | | | 4a | 1, 2, 4, 5, 7, 8 |
| 7a | 3.48 | 10.2 | 75.6 | | 4, 5, 6, Hex-1' |
| 7b | 3.78 | 10.2 | | | 4, 5, 6, Hex-1' |
| 8 | 3.68 | s | 61.8 | | 2, 8 |
| **Thr** | | | | | |
| 9 | 4.05 | d, 4.7 | 67.3 | 3 | 1, 10, 11, 12 |
| 10 | - | | 177.7 | | - |
| 11 | 4.31 | dd, 4.7, 6.5 | 70.5 | 2, 4 | 10, 11, 12 |
| 12 | 1.26 | d, 6.5 | 22.0 | 3 | 9, 11, 12 |
| **α-D-Glc** | | | | | |
| 1' | 4.95 | d, 3.7; $^{1}J_{C1, H1}$ = 170 | 101.3 | 2' | 1', 2', 3', 5' |
| 2' | 3.58 | 3.6, 9.9 | 74.0 | 1', 3' | 3' |
| 3' | 3.74 | | 75.5 | 2', 4' | 1', 2', 4' |
| 4' | 3.41 | 9.3, 9.6 | 72.1 | 3', 5' | 3', 5', 6' |
| 5' | 3.63 | 2.2, | 74.8 | 4', 6a', 6b' | 1', 3', 4' |
| 6a' | 3.75 | | 63.1 | 5', 6b' | 1', 5', 6' |
| 6b' | 3.85 | 2.2, 12.2 | | 5', 6a' | 1', 4', 5', 6' |

* = Exchangeable signals

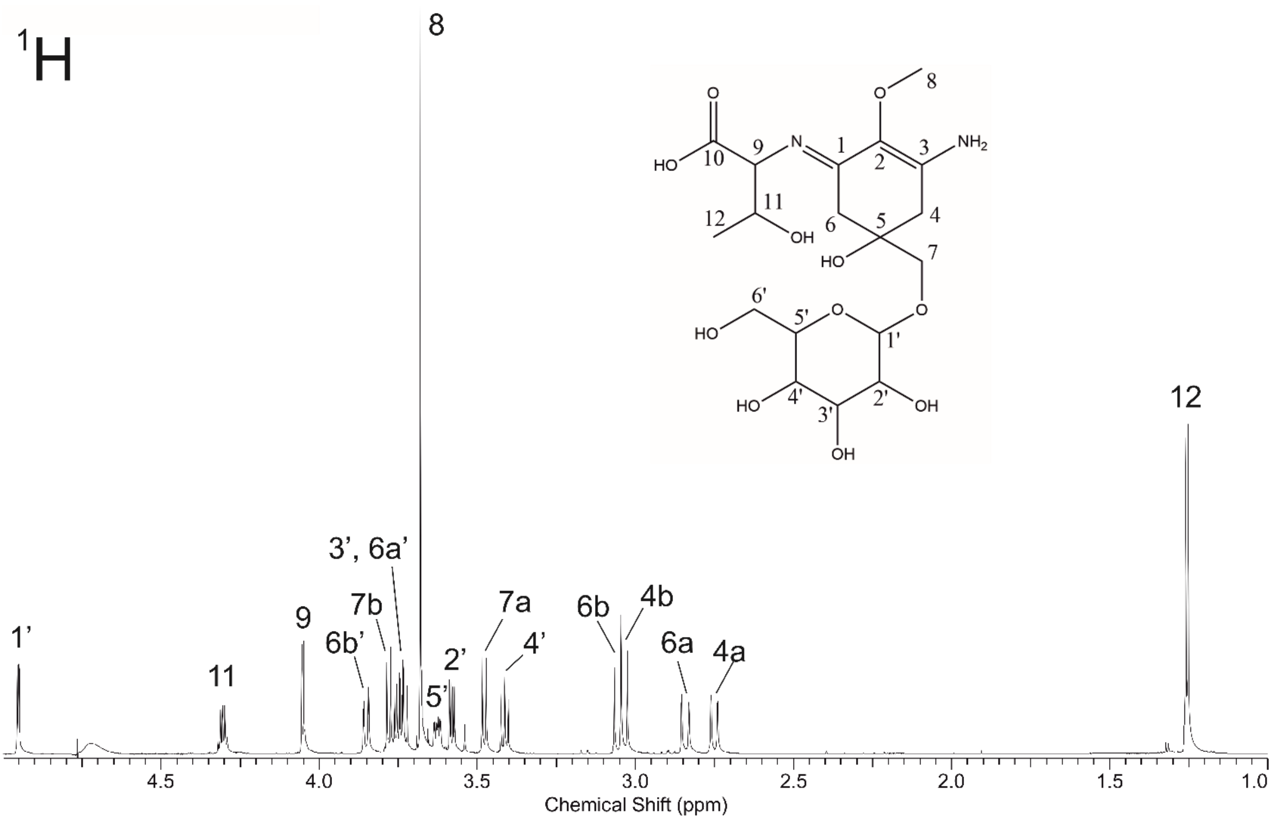


**Figure S13.** Annotated proton spectrum of 450 Da MAA in $D_2O$.

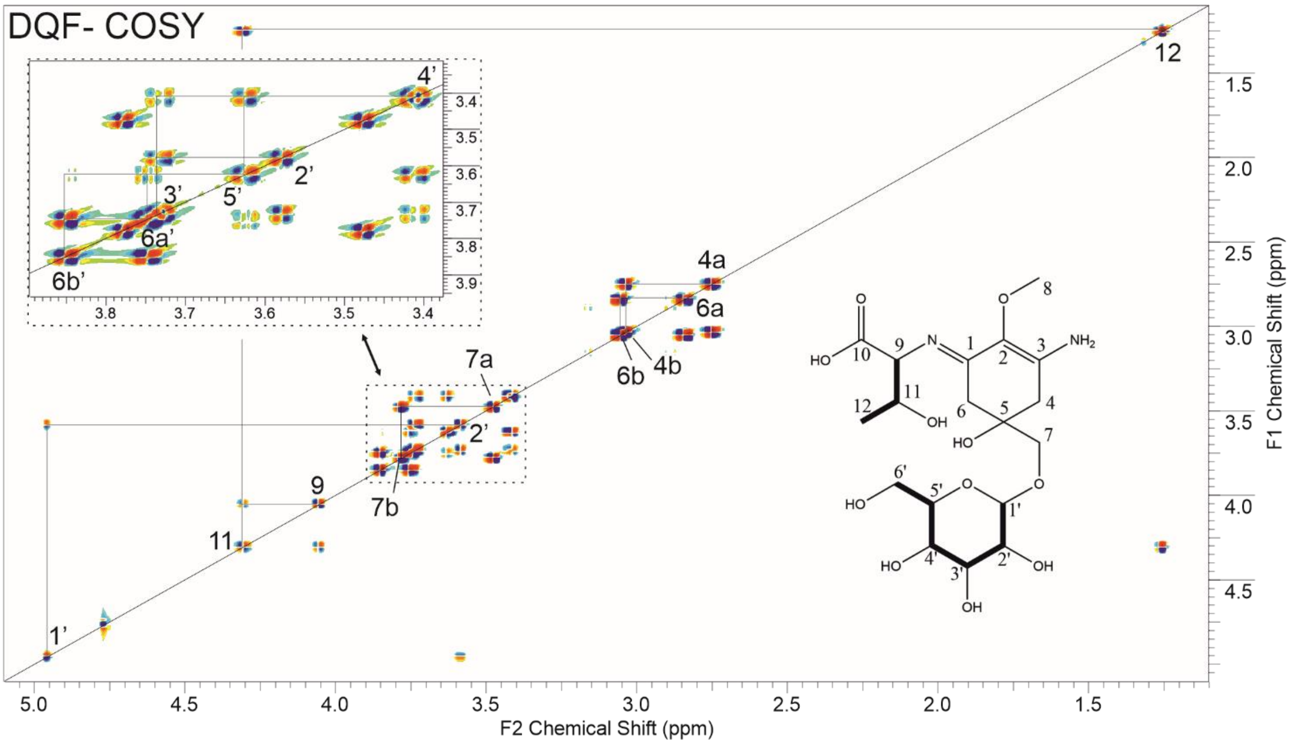


**Figure S14.** Annotated $^{1}H$-$^{1}H$ DQF-COSY spectrum of 450 Da MAA in $D_2O$. COSY correlations marked with bold bonds to the structure.

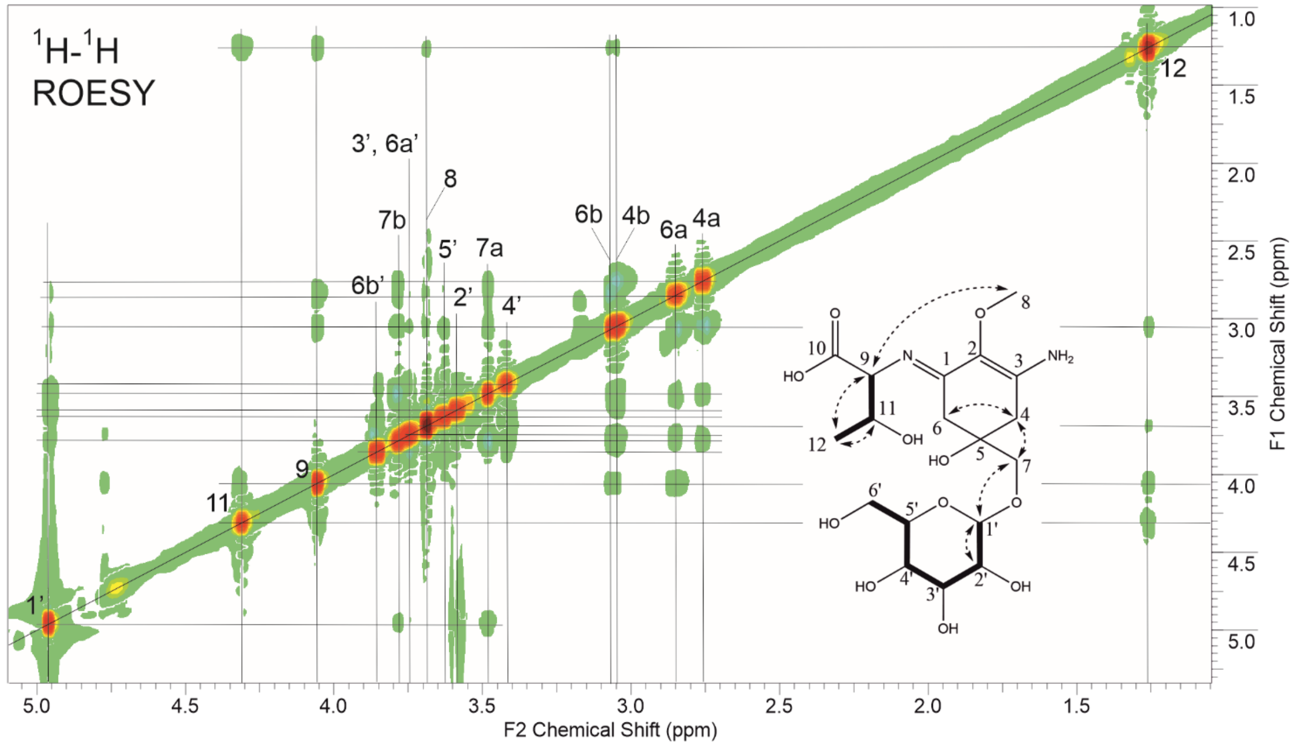


**Figure S15.** Annotated ${}^{1}H$-${}^{1}H$ ROESY spectrum of 450 Da MAA in $D_2O$. ROESY correlations marked with dashed arrows to the structure and COSY correlations with bold bonds.

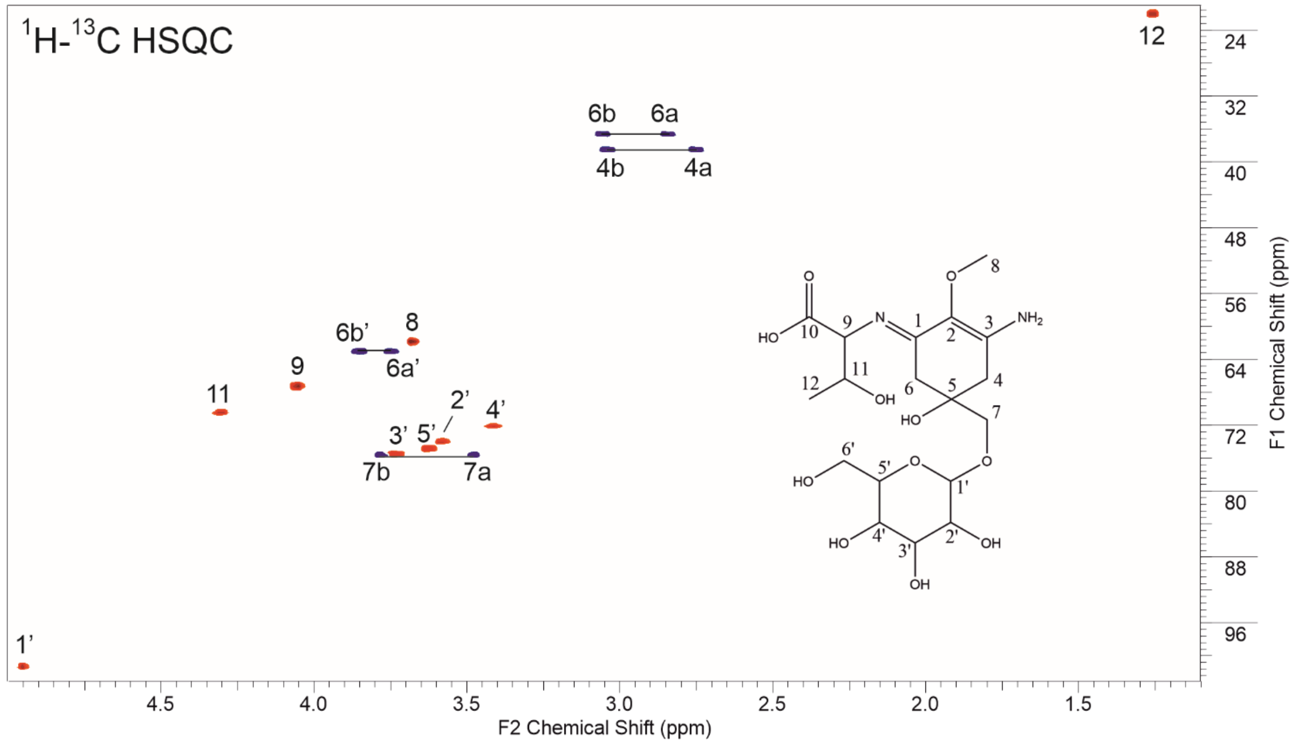


**Figure S16.** Annotated edited ${}^{1}H$-${}^{13}C$ HSQC spectrum of 450 Da MAA in $D_2O$.

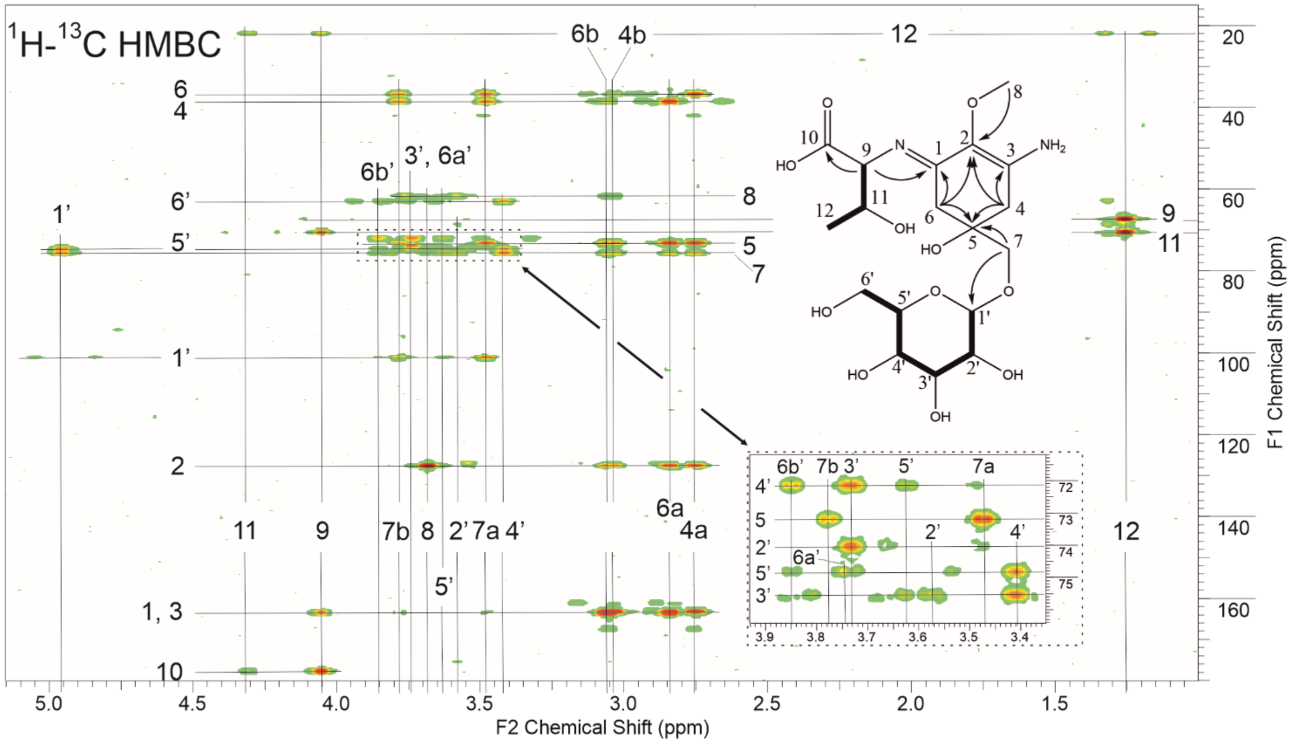

**Figure S17.** Annotated $^{1}H$-$^{13}C$ HMBC spectrum of 450 Da MAA in $D_2O$. HMBC correlations marked with arrows and COSY correlations with bold bonds to the structure.

**Table S7.** Complete genome assembly stats for *Nostoc* sp. UHCC 0302 (CP151099).

| DNA | Size (bp) | Coverage |
|---|---|---|
| Chromosome | 8388664 | 65 |
| Plasmid 1 | 383299 | 48 |
| Plasmid 2 | 358430 | 63 |
| Plasmid 3 | 282759 | 71 |
| Plasmid 4 | 40344 | 23 |
| Plasmid 5 | 37423 | 24 |
| Plasmid 6 | 33907 | 18 |
| Plasmid 7 | 31762 | 25 |
| Plasmid 8 | 30468 | 16 |

**Table S8.** MAA biosynthetic enzymes of *Nostoc* sp. UHCC 0302 (CP151099) as annotated and deposited in GenBank.

| Gene name | Protein ID | Size (aa) | Product |
|---|---|---|---|
| *mysA* | WZF19242.1 | 409 | Sedoheptulose-7-phosphate cyclase |
| *mysB* | WZF19243.1 | 277 | Class-I SAM-dependent methyltransferase |
| *mysC* | WZF19244.1 | 465 | ATP-grasp domain containing protein |
| $mysD_1$ | WZF19246.1 | 348 | Mycosporine-glycine-alanine ligase |
| *mysH* | WZF19248.1 | 297 | Phytanoyl-CoA dioxygenase |
| $mysD_2$ | WZF19974.1 | 345 | Mycosporine-glycine-alanine ligase |
| $mysJ_1$ | WZF19245.1 | 296 | beta-1,6-N-acetylglucosaminyltransferase |
| $MysG_1$ | WZF19247.1 | 432 | Glycosyltransferase family 4 protein |
| $mysJ_2$ | WZF19973.1 | 304 | beta-1,6-N-acetylglucosaminyltransferase |
| $MysG_2$ | WZF19972.1 | 423 | Glycosyltransferase family 4 protein |

**Table S9.** Primers used for the cloning of MAA biosynthetic gene clusters of *Nostoc* sp. UHCC 0302.

| **Primer Name** | **5'- 3' Nucleotide Sequence** |
|---|---|
| P1(*mysA*_fwd) | CATGCCATGGGGTACCGAACCCAAGCAAAATATAATAAAAGGAGGTTTTT TATGAATAATGTTCAAGCATCGT |
| P2 (*mysD*_rev) | CGCGGATCCGCTAGCTCAATTCTGCAACACCTTTT |
| P3 (*mysC*+p_rev) | GTGCTCGAGTGCGGCCGCAAGCTTGTTAATCGCCATCTAATTCC |
| P4 (*mysJ*+p_rev) | GTGCTCGAGTGCGGCCGCAAGCTTGCTAGGATGTAATCTTATCAAGTTCA TTAAGAATATTG |
| P5 (*mysH*_fwd) | TTGCAGAATTGAAGCGTTCAACATAGCACAG |
| P6 (*mysG*_rev) | GTGCTCGAGTGCGGCCGCAAGCTTGTCAAAAGGTTGTTAATTCATAG |
| P7 (*mysH*+p_rev) | GTGCTCGAGTGCGGCCGCAAGCTTGTTAGCAATGCAGTTCAACTC |
| S1 (T7 term rev) | GCTAGTTATTGCTCAGCGG |
| S2 (mysC_S_fwd) | CTGCCTCAATCTACAATGCC |
| S3 (mysD_S_fwd) | CACAAAGCCAGAGCCAAAG |
| S4 (mysH_S_fwd) | GACTGACTGCTTGTGAATCC |
| S5 (pET_S_fwd) | ATGCGTCCGGCGTAGA |
| X1(araBAD _ Fwd) | CTGACGCTTTTTATCGCAACTCTCTACT |
| X2 (t7_terminator_rev) | GATTTGTCCTACTCAGGAGAGCGTTCA |
| X3 (mysA_S_rev) | CACTTGGCTATTCGCAACCAC |
| X4 (mysH_S_fwd) | CTACTGGCCGATCCAGGG |
| X5 (mysC_S_fwd) | CTGCTGAAGAATCTGCAGC |
| X6 (mysD1_S_fwd) | CGAGCCGTGGTTCCTGGAAGC |
| X7 (mysD2_S_fwd) | GCCTGTTCGACTTTCGCATCG |

**Table S10.** RBS sequences calculated for each MAA biosynthetic gene cluster enzyme of *Nostoc* sp. UHCC 0302. (*We did not calculate an individual RBS sequence for MysB as it was almost coupled with the MysA enzyme with only 4 bases in between.)

| **Enzyme** | **RBS sequence 5' to 3'** |
|---|---|
| MysAB* | ATTCCTGCACAAGCGGGCACAGAGAAATTAAGGAGGTTTTTT |
| MysC | GGGGATATTCCCTCTTCACAATACTAAACCCTAAGGAGGTATTTT |
| MysD$_1$ | TTGTTGTAATAAGTTCGCAGGAAGCTGCGATTCGGGGAGGTTTTTA |
| MysD$_2$ | GGTTTGGTCCTTGTTCAGTTCGTTAAGGAGGTTTTTA |
| MysH | CCCCCAACAACGTAGGCGGGTCAGACCCGTATAAGGAGAATTTATT |
| MysJ$_1$ | ATTATCATAACAGGATACTAAGGAGGTCAGA |
| MysJ$_2$ | CCAGCCCTTGTACTGGGCGCATCATCGAGTAAGGAGGTAAGT |
| MysG$_1$ | TCGCGAGAAAAAGGAGGTCGAAT |
| MysG$_2$ | CCATCAAGAGAATAGCGCTCTTATATCCCAAAAATTAAGGAGGTTTTTTT |

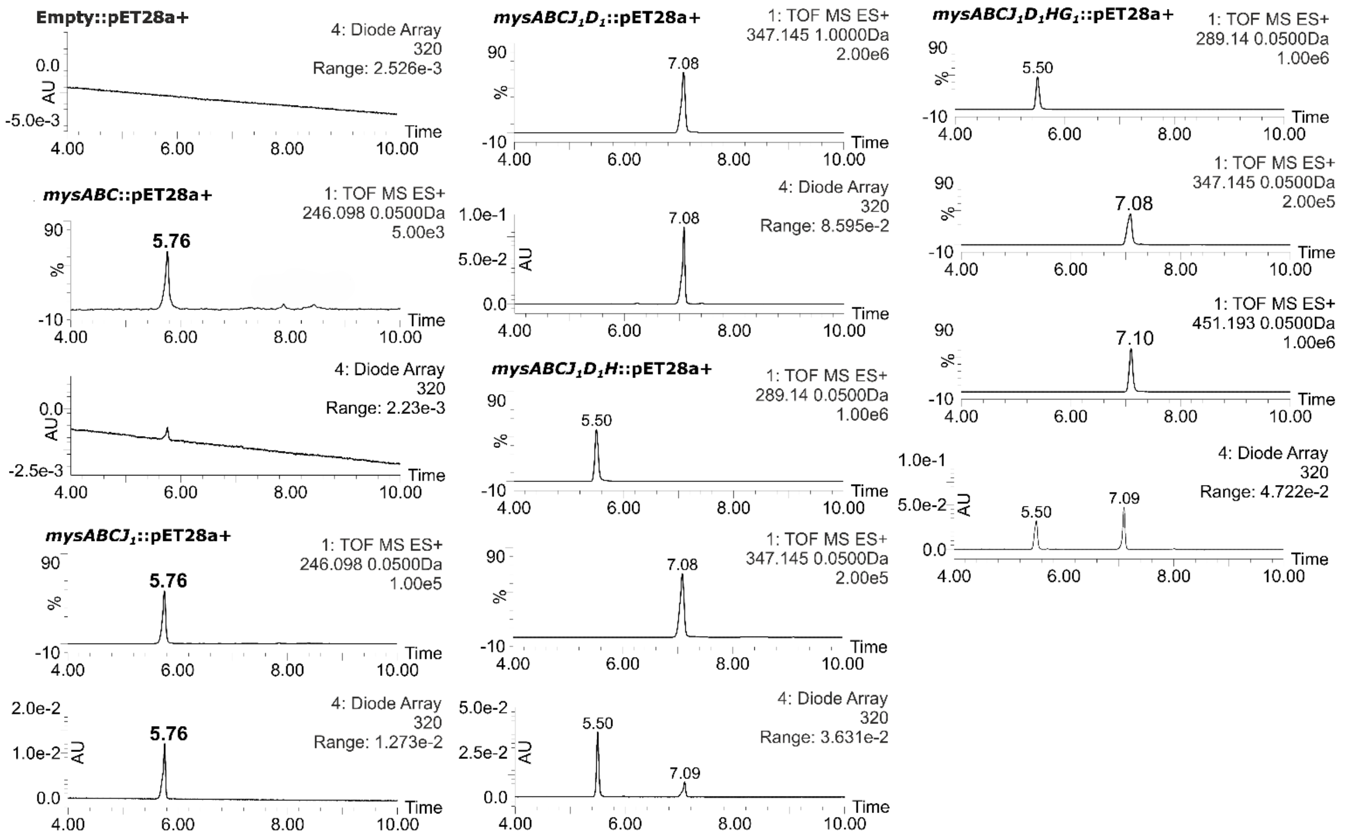


**Figure S18.** MS ES+ and UV chromatograms of different MAA variants detected from the extracts of recombinant *Escherichia coli* BL21 (DE3) cultures expressing different MAA biosynthetic gene cluster constructs from *Nostoc* sp. UHCC 0302 in pET28a+.

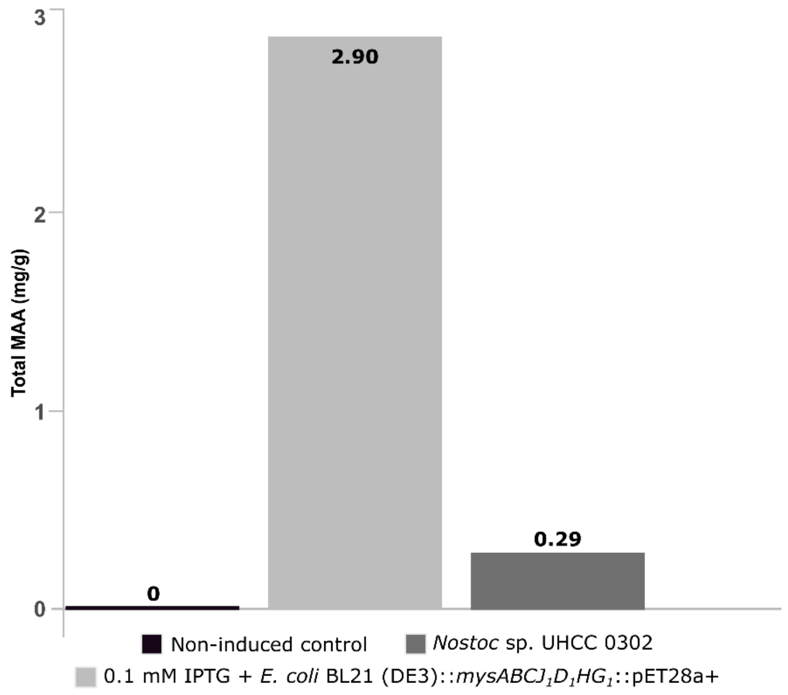


**Figure S19.** Total MAA concentration per dry biomass (mg/g) over 24 hours of 0.1 mM IPTG induced and non-induced production in recombinant *E. coli* clones expressing the *mysABCJ$_1$D$_1$HG$_1$* gene cluster in pET28a+ system in LB medium supplemented with 1 % Xylose, versus the 30-day old cultures of *Nostoc* sp. UHCC 0302 grown in optimal laboratory growth conditions.

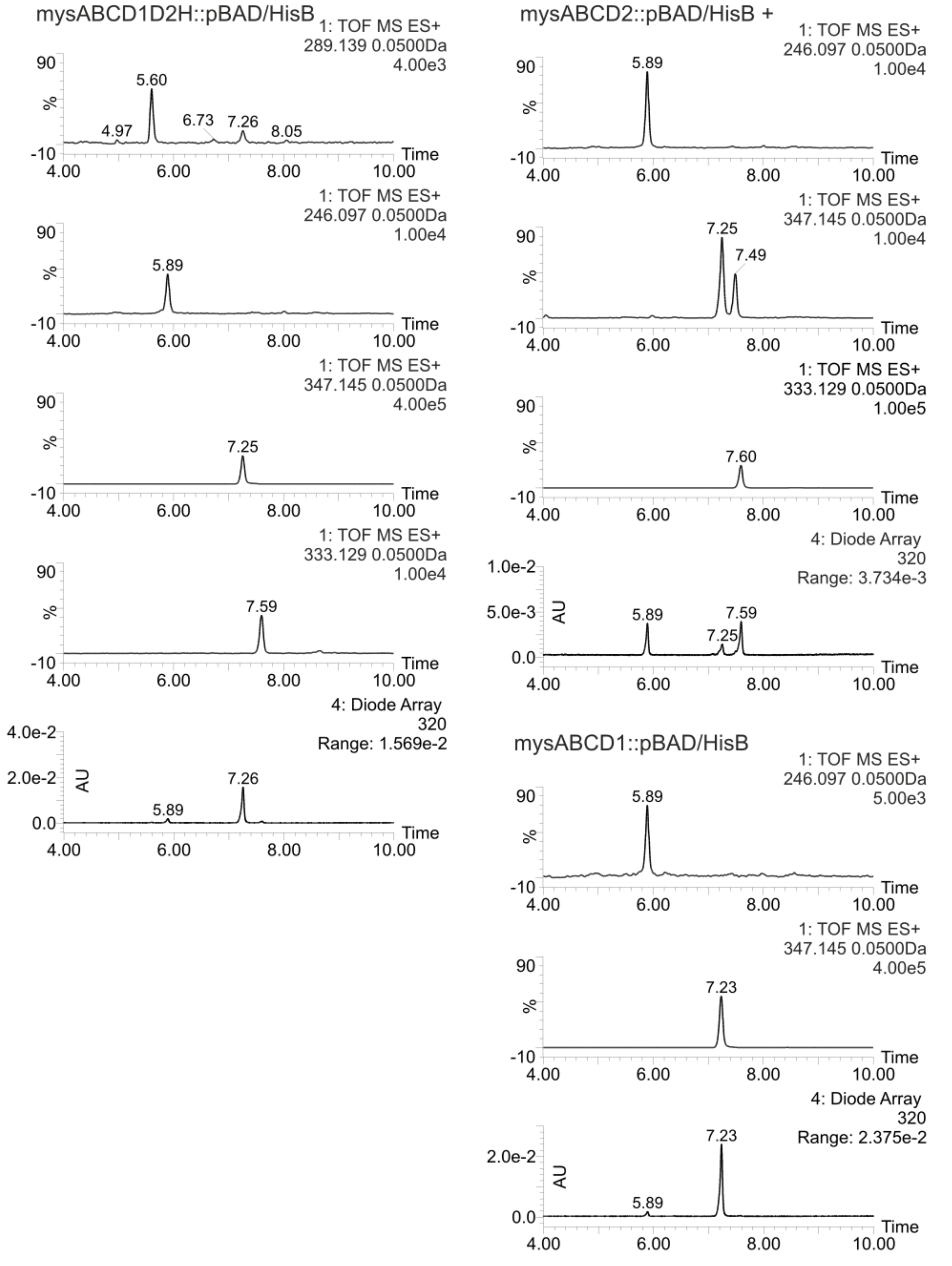


**Figure S20.** MS ES+ and UV chromatograms of different MAA variants detected from the extracts of recombinant *Escherichia coli* BL21 (DE3) cultures expressing different synthetic constructs of MAA biosynthetic gene cluster genes from *Nostoc* sp. UHCC 0302 in pBAD/His B.

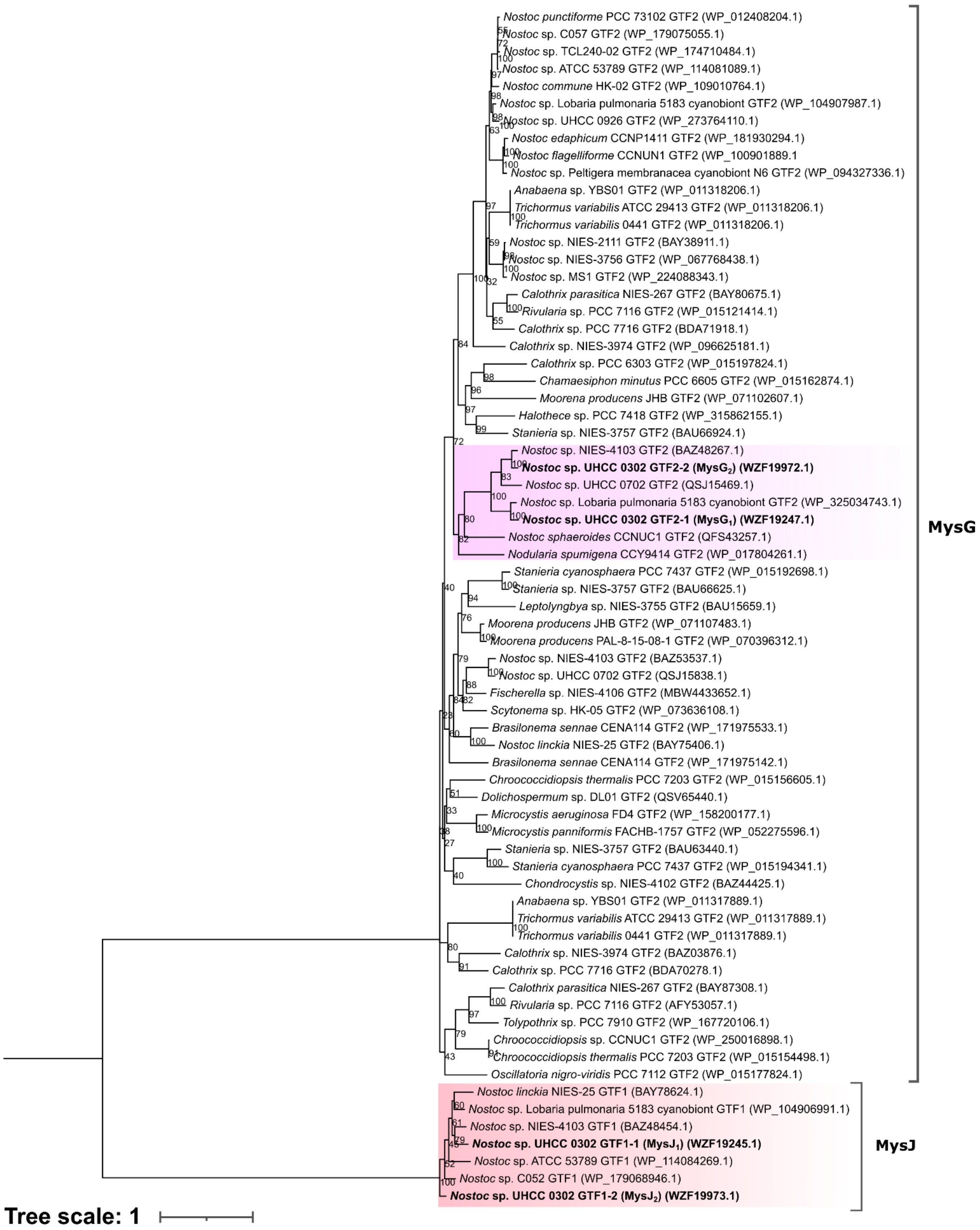


**Figure S21.** Phylogenetic distribution of MysJ and MysG in 52 complete cyanobacterial genomes containing a MAA biosynthetic gene cluster. Phylogenetic tree constructed using amino acid sequences of the glycosyltransferase enzymes using IQ-TREE v1.7 with the substitution model of JTTDCMut+F+I+G4. (GTF: Glycosyltransferase, GTF1: MysJ, GTF2: MysG

**Table S11.** Homology based protein structure prediction summary using Swiss-Model[1] for *Nostoc* sp. UHCC 0302 MysJ1/2 and MysG1/2 enzymes. NAG: 2-acetamido-2-deoxy-beta-D-glucopyranose. UDX: uridine-5'-diphosphate-xylopyranose. GLC-F6P: 6-O-phosphono-beta-D-fructofuranose-(2-1)-alpha-D-glucopyranose. UDP: uridine-5'-diphosphate. *Automatic entry from UniProt, not reviewed.

**MysJ$_{1/2}$ Hits:**

| Code | J1/J2 Seq. Identity (%) | Method | Ligands | Oligo State | Name | Organism | Reference |
|---|---|---|---|---|---|---|---|
| A0A2A2TLA8.1.A | 80.74 / 78.55 | AlphaFold v2 | uncharacterized | monomer | Peptide O-xylosyltransferase | *Calothrix elsteri* CCALA 953 | Uniprot Entry: A0A2A2TLA8 · A0A2A2TLA8_9CYAN |
| 6ej7.1.A | 31.71 / 28.83 | X-ray, 2.0 Å | 1 x NAG, 1 x UDX | Hetero-dimer | Xylosyltransferase 1 | Human | Briggs & Hohenester (2018) - doi.org/10.1016/j.str.2018.03.014 |

**MysG$_{1/2}$ Hits:**

| Code | G1/G2 Seq. Identity (%) | Method | Ligands | Oligo State | Name | Organism | Reference |
|---|---|---|---|---|---|---|---|
| A0A1Z4S0N4.1.A* | 70.12 / 90.74 | AlphaFold v2 | uncharacterized | monomer | A0A1Z4S0N4 | *Nostoc* sp. NIES-4103 | Accession (BAZ48267.1) |
| 6kih.1.A | 21.05 / 22.07 | X-ray, 3.0 Å | 1 x GLC-F6P, 1 x UDP | monomer | Sucrose-phosphate synthase (tll1590) | *Thermosynechococcus elongatus* | Li et al., (2020) - doi.org/10.3389/fmicb.2020.01050 |

*1 Waterhouse, A. et al. SWISS-MODEL: homology modelling of protein structures and complexes. Nucleic Acids Res 46, W296–W303 (2018).*
